# Supplementary material for: Gold-nanofève surface-enhanced Raman spectroscopy visualizes hypotaurine as a robust anti-oxidant consumed in cancer survival
Source: Nat Commun. 2018 Apr 19;9:1561. doi: 10.1038/s41467-018-03899-1 (PMC5908798; doi:10.1038/s41467-018-03899-1)
Supplement: Supplementary file 1 — Supplementary Information [file 41467_2018_3899_MOESM1_ESM.pdf]

SUPPLEMENTARY INFORMATION

Shiota M, et al.

Gold-nanofève surface-enhanced Raman spectroscopy visualizes hypotaurine as a robust anti-oxidant consumed in cancer survival

Supplementary Note 1

(Reference numbers follow those for main text)

Characterization of gold-nanofève (GNF) substrate
(For Supplementary Figure 1)

At the deposition angle of 80°, rhodamine 6G (R6G)-derived SERS signals [17] became significantly greater under the polarized light in parallel with the direction of Au deposition for GNF substrates (Parallel-polar in **Figure 1c** and **Supplementary Figures 1a and 1b**) than those induced by the perpendicularly polarized light (Perpendicular-polar in **Figure 1c** and **Supplementary Figure 1a and 1b**). As a function of Au-thickness, GNF-SERS signal intensities at 1360 cm^{-1} for R6G displayed the maximal intensity at 110 nm in Au thickness (**Supplementary Figure 1c**), suggesting that greater Au deposition to facilitate growth of Au-nanoparticles does not necessarily induce further SERS enhancement. Averaged intensities among 3 different lots of GNF substrates showed high reproducibility as the coefficient of variation was 2.1%. SERS imaging of R6G at 1360 cm^{-1} indicated an optimal uniformity of large-area imaging using GNF substrates, as judged by the coefficient of variation of 11.0% ± 0.5% (mean ± SE of 3 different production lots); the value was comparable to that in GNC substrates as reported previously (**Supplementary Figures 1d and 1e**) [15].

Characterization of SERS signals for thiols and hypotaurine
(For Supplementary Figures 4 and 5)

SERS signals of varied thiol metabolites were examined on GNF substrates through sufficient concentrations of analytes. These measurements utilized natural-dried water solutions, and the relationship of the original concentrations of analytes to the analyte densities on GNF substrates is described in **Supplementary Figure 4a**. When the analyte density was 159.2 pmol mm^{-2}, the peaks of reduced (GSH) and oxidized glutathione (GSSG) occurred at 298 cm^{-1} (**Supplementary Figure 4**). Ophthalmic acid, in which a cysteine residue of GSH is replaced with 2-aminobutylate [23], generated no detectable signal at 298 cm^{-1}, indicating that the cysteine residue of GSH is responsible for Au-S binding. We also examined if HS^- at 50 μmol l^{-1}, which is greater than physiological concentrations in livers [24], and other sulfur-containing metabolites such as hypotaurine (HT) and taurine (**Supplementary Figure 4a**), but observed no notable signal at 298 cm^{-1}. In contrast, bovine serum albumin (BSA), which has one reactive cysteine residue, did not generate any detectable GNF-SERS signal at a physiologically relevant concentration (**Supplementary Figure 4a**). Previous Raman studies suggested that retinol yields peaks at 1150 cm^{-1} [25]. As seen in Suppl. Fig. 4a, retinol and its storage form, retinol palmitate,

and β-carotene, a precursor of retinol, generated SERS peaks at 1150 cm^{-1}. Given that retinol and retinol palmitate are mainly stored in hepatic stellate cells and partly stored in hepatocytes [26, 27], the results in **Figure 3d** suggest that GNF-SERS peak occurring predominantly in parenchyma at 1150 cm^{-1} corresponds to these retinoid metabolites.

As seen in Suppl. Fig. 4, SERS signals of HT appeared to be overlapped with those of retinol palmitate. We determined contents of retinol palmitate, retinol and β-carotene in the control livers of B6 mice by a method shown later in **Methods of Supplementary Note**. The hepatic contents of retinol palmitate were 514.8 ± 101.3 μg g^{-1} (mean ± SEM of 6 separate experiments. Molecular weight =524.8 that gives 0.98 mmol l^{-1}), while both retinol and β-carotene were undetectable under given experimental conditions. Careful comparison of SERS spectra between HT and retinol palmitate at 1 g l^{-1} in methanol led us to conclude that 978 ± 8 cm^{-1} is the best wave number for HT detection to minimize contamination of retinol palmitate peaks (**Supplementary Figure 4b**). In the control brain samples, contents of retinol palmitate were 5.9 ± 0.8 μg g^{-1} (mean ± SEM of 5 separate experiments, while free retinol and β-carotene were undetectable

We then constructed dose-responsive curves of GSH for the SERS signals that reached a plateau level at the analyte density of 5 pmol mm^{-2}, suggesting saturation of the analyte on GNF substrates. On the other hand, the SERS signal of GSSG occurred at the same wave number at 298 cm^{-1}, and was weaker than GSH. However, Au-nanoparticles can execute disulfide cleavage [28] and thus GSSG appears to bind to Au surface as a form of GS^-, suggesting that the peak at 298 cm^{-1} reflects GSH (**Supplementary Figure 4**). Of note, metabolites including homocystine (Hcy-Hcy), homocysteine (Hcy), cystine (Cys-Cys) and cysteine (Cys) in a range of analyte densities between 0 and 5 pmol mm^{-2}, yielded a SERS peak at 298 cm^{-1} (**Supplementary Figure 5b**). None of these analytes yielded SERS signals at 978 cm^{-1} (**Supplementary Figures 5a and 5b**). In contrast, HT, but not taurine, generated a peak at 978 cm^{-1} but not at 298 cm^{-1} (**Supplementary Figure 5c**).

MALDI imaging induces auto-oxidation of thiols and HT
(For Supplementary Figures 6-10)

Capillary electrophoresis-mass spectrometry (CE-MS) (**Supplementary Table 2**) revealed that glutathione contents in tumour-bearing livesrs are approximately several μmol g^{-1}, while those of Hcy-Hcy, Hcy, Cys-Cys and Cys are around several nmol g^{-1}, being 2- or 3-order smaller than contents of glutathione and taurine. GSH, Hcy and Cys are prone to oxidation by MALDI under the atmospheric pressure (AP) conditions (**Supplementary Figure 5)**, and are converted artificially towards sulfonated compounds including glutathione sulfonate (GSO_3^-), homocysteic acid, cysteic acid, respectively. Likewise, AP-MALDI-IMS caused oxidation of HT

into taurine (**Supplementary Figures 5 and 6**). To exclude involvement of these disulfides and thiols in GNF-SERS signals at 298 cm^{-1} in vivo (**Figure 4a**), we took advantage of AP-MALDI to examine whether these metabolites are detectable in normal and tumour-bearing livers [12, 29]; strict gating analyses of the individual mass peaks (**See Methods**) revealed that disulfides (Hcy-Hcy and Cys-Cys) and the corresponding thiols (Hcy and Cys) or their terminal sulfonates (homocysteic and cysteic acids) were undetectable under AP-MALDI conditions. These results suggested that amounts of parent disulfides or thiols or sulfinates are few, if any, in normal and tumour-bearing livers (**Supplementary Figures 7a-7c**). Data collected from CE-MS analyses (**Supplementary Table S2**) confirmed that tissue contents of GSO_3^-, homocysteic acid, or cysteic acid are measurable in tumour-bearing livers (**Supplementary Figure 8**). These results suggest that artificial oxidation of reduced forms of metabolites is inevitable in both AP-MALDI and CE-MS.

As previously reported [29], MALDI-IMS enabled to detect GSH, glutathione sulfonate (GSO_3^-), and GSSG in the control liver, and these mass peaks were enhanced in tumour-bearing livers (**Supplementary Figure 9a**). These peaks occurred predominantly in metastasized tumours (TB in **Supplementary Figure 9b**): Distribution of GSH and its oxidized derivatives matched that of SERS imaging at 298 cm^{-1} (**Supplementary Figure 9b**). These results suggest that glutathione (GS), but neither cyst(e)ine nor homocyst(e)ine, serves as a putative metabolite visualized by SERS at 298 cm^{-1} (**Figure 3c**).

HT was undetectable in AP-MALDI-MS spectroscopy in control and tumour-bearing livers (**Supplementary Figures 8a-8d**). Under such circumstances, taurine is markedly elevated in both tumour and parenchyma in tumour-bearing (TB) livers, raising a possibility that HT is auto-oxidized into taurine under the AP-MALDI conditions. Considering that taurine is a terminally-sulfonated metabolite derived from cysteamine and HT (**Supplementary Figure 6**), we hypothesized that HT is readily oxidized into taurine, when the metabolites are ionized under the atmospheric O_2 pressure. To test this hypothesis, vacuum-type MALDI-IMS was used for analyses [30]. The standard HT in vitro generated secondarily oxidized taurine under the lowest laser magnitude (40% in **Supplementary Figure 10a**). The increasing laser power did not linearly increase the HT peak, while the taurine peak was increased: pie charts showing the ratio of HT versus taurine was markedly diminished when the laser power became greater than 60%, suggesting that laser exposure causes marked HT oxidation; the event is consistent with MALDI-induced sulfonation of the standard HT (**Supplementary Figure 7d**). Under the 40% laser power, vacuum-type MALDI-MS and -MS^2 imaging enabled to visualize HT in parenchyma, but not in tumours (**Supplementary Figures 10b and 10c**).

GNF-SERS spectral library of liver metabolites

We examined GNF-SERS spectra of major metabolites in the liver: While NH_4^+, urea, SO_3^{2-}, creatinine, phosphatidyl choline and taurocholate [31] exhibited GNF-SERS peaks, these peaks did not overlap SERS signals at 298 cm^{-1}, 978 cm^{-1} and 1150 cm^{-1} (**Supplementary Figures 11a and 11b**). Furthermore, Hcy-Hcy, Hcy, Cys-Cys and Cys that exhibited SERS signals at their supra-physiological concentrations (**Supplementary Figure 5b**) did not show any notable signals at 298 cm^{-1} at 10 μmol l^{-1} (**Supplementary Figure 11c**), a physiologic concentration of these metabolites (< 10 μmol l^{-1}) judged from metabolomics data in **Supplementary Table 2**.

Effects of SSZ and 2-DG on ROS levels in cancer cell lines

Many cancer cell lines expressing CD44 stop proliferation or lose viability by stable knockdown of CD44. We investigated 5 different cancer cell lines (**Figures 7** and **8**, and **Supplementary Figure 14a**).; MCF7 (CD44-low breast cancer) and MDA-MB-231 (CD44-high breast cancer), and CD44v-positive non-small cell lung carcinoma cell lines such as PC9, H1650 and H2170. These cells express ADO and CDO, and 3 key enzymes such as PHGDH, PSAT1 and PSPH, the gate-keeping enzymes for providing glycolytic substrates towards serine and glycine-cleavage systems, with expressing SHMTs and glycolytic enzymes. Among 5 different cancer cell lines, MCF7 and MDA-MB-231 exhibited SSZ-dependent ROS elevation, and co-incubation with 2-DG dose-dependently increased ROS. Such elevation of ROS by SSZ was undetectable in PC9. On the other hand, in the absence of 2-DG, H1650 displayed SSZ-induced ROS increases, but did not show 2-DG-dependent ROS elevation (**Supplementary Figure 14b**). Taking these results into accounts, MCF7 and MDA-MB-231 are considered cell lines exhibiting comparable responsiveness of ROS to SSZ and 2-DG analogous to HCT116 cells (**Supplementary Figure 13**). As seen in **Supplementary Figure 14c**, 2-DG-induced ROS enhancement was dose-dependently attenuated by exogenously administered HT.

Supplementary Methods

MALDI imaging mass spectrometry (MALDI-IMS)

In separate sets of experiments, we conducted imaging MS according to our previous methods [12, 29, 66]. Serial sections used for SERS imaging were used for AP-MALDI-IMS according to our previous methods [12, 29, 66]. Briefly, as a matrix, 5 mg ml^{-1} 9-aminoacridine (Merck Schuchardt, Hohenbrunn, Germany) in 80% ethanol solution was sprayed over the 5-μm thickness liver sections which were thaw-mounted on ITO-coated glass slides (Matsunami Glass IND., LTD, Osaka, Japan). AP-MALDI-IT-TOF mass spectrometer (Shimadzu Corp., Kyoto,

Japan) allowed us to collect the data in the negative ion mode with the recording mass range from *m/z* 300 to 670. After selecting the region of interests (ROIs) by light microscopic observations, a series of repeated laser irradiation at 100 times per spot was performed at 12-µm pitch intervals at 166 x 166 spots, giving 35,344 data points in total for each scan. Mass spectra from each measuring spot were analyzed and peaks of interest were extracted by house-made software SIMtools (Shimadzu Imaging Mass spectrometry toolbox for MATLAB(R)). To evaluate small molecules such as taurine, we performed the measurement with the mass range of *m/z* 100‑325 at 12-µm intervals at 166 x 166 spots. Mass peaks of metabolites collected from tissue sections were identified by comparing MS/MS fragment patterns with those collected from standard reagents. To determine and visualize specific mass peaks of Hcy-Hcy, Hcy, Cys-Cys and Cys, and their sulfonated end products homocysteic acid and cysteic acid, the *m/z* values of their specific mass fragments were gated within a range of *m/z* ± 0.01 to minimize noise signals (**Supplementary Figures 7 and 8**). The same system was used to examine coronal sections collected from frozen tissue blocks of glioma-bearing brain tissues, when necessary.

Since AP-MALDI caused auto-oxidation of metabolites such as thiols and HT, we examined whether vacuum-type MALDI imaging serves as an alternative method to minimize oxidative degradation of metabolites. Briefly, the 5-µm sections of the tumour-bearing livers were prepared with a cryo-microtome (CM3050, Leica Microsystems). Sections were attached onto indium-tin-oxide (ITO)-coated glass slides (Bruker Daltonics). Those sections were coated with 9-aminoacridine as the matrix (10 mg/ml, dissolved in 80% ethanol) by manually spraying with an artistic-brush (Procon Boy FWA Platinum, Mr. Hobby). The matrix was simultaneously applied to the multiple sections to maintain consistent analyte extraction and co-crystallization conditions. MALDI imaging was performed using both, Ultraflextreme MALDI-TOF/TOF mass spectrometer equipped with an Nd:YAG laser and linear ion trap MS with a MALDI source (MALDI LTQ XL, Thermo Fisher Scientific Inc.) equipped with a nitrogen laser (337 nm; 60 Hz) [30]. In the TOF/TOF measurement, data were acquired in the negative reflectron mode and signals between m/z 50 and 1000 were collected. Each spectrum was the result of 300 laser shots at each data point and pitch distance of raster scanning was 70 µm. The laser power was optimized to minimize in-source decay of HT (set at 40%, as described in **Supplementary Figure 10**). In the linear ion trap MS measurement, the laser energy and the raster step size were set at 25 µJ and 75 µm, respectively. During imaging measurements, specific ion transitions for HT (*m/z* 108→65) and ATP (*m/z* 506→408) were monitored with a precursor ion isolation width of 1.0 *m/z* units. The obtained spectral data were then transformed to image data using ImageQuest 1.0.1 software (Thermo Fisher Scientific Inc.).

Cancer cell lines and western blot analyses

Besides HCT116 human colon cancer cell lines, we conducted in-vitro experiments using several different cancer cell lines derived from human such as MCF7 that displays little CD44 expression, and MDA-MB-231, displaying CD44 (breast cancer cells), and PC9, H1650 and H2170, non-small cell lung cancer cell lines expressing CD44v and resistant to sulfasalazine, an inihibitor of xCT (**Supplementary Figures 14a and 14b**). These cell lines were used to examine whether these cells express PHGDH, PSAT1 and PSPH to protect against oxidative stress through the action of hypotaurine. To detect CD44, the cell extracts were mixed with equivalent volume of 2 x Laemmli sample buffer (62.55 mmol l^{-1}, Tris-HCl; pH6.8, 2% SDS, 25% Glycerol, and 0.01% Bromophenolblue) with or without 10% (w/v) 2-mercaptoethanol. The equivalent amounts of proteins (25 μg/lane) were separated by 10% SDS-PAGE and transferred onto PVDF membranes. The membranes were blocked in PBST (0.15 mol l^{-1}, NaCl, 10 mmol l^{-1} Tris-HCl; pH 7.5, and 0.1% Tween 20) containing 3% (w/v) skim milk for 60 min at room temperature. Subsequently, the membranes were probed with the primary antibody for overnight at 4°C. Following the incubation of primary antibodies, the membranes were incubated with the appropriate HRP-conjugated secondary antibody for 2 hrs at room temperature. Signals were visualized by ECL Prime (GE Healthcare, Amersham, UK). The following primary antibodies were used in Western blotting: anti-CD44 mouse monoclonal (ab6124, Abcam, Cambridge, UK; 3,000 x dilution), anti-CSE mouse monoclonal (H00001491-M03, Abnova, Taipei, Taiwan; 7,000 x dilution), anti-CBS mouse monoclonal (H00000875-M01, Abnova; 7,000 x dilution), anti-beta actin mouse monoclonal (A1978, Sigma-Aldrich, St. Louis, MO; 10,000 x dilution). Following antibodies were also used for Western blot analyses of shControl and shCD44 cells; anti-PHGDH rabbit monoclonal (#66350S, Cell Signaling: 5,000 x dilution), anti-PSAT1 rabbit polyclonal (HPA042924, SIGMA; 3,000 x dilution), anti-SHMT1 rabbit monoclonal (#80715S. Cell Signaling; 5,000 x dilution), anti-SHMT2 rabbit polyclonal (#12762, Cell Signaling; 5,000 x dilution), anti-PGK1 goat polyclonal (sc-17943, Santa Cruz biotechnology, Dallas, TX; 3,000 x dilution), anti-ENO1 goat polyclonal (sc-7455, Santa Cruz; 3,000 x dilution), anti-phospho PKM2(S37) rabbit polyclonal (11456, Signaling antibody, Baltimore, MD; 2,500 x dilution), anti-PKM2 rabbit monoclonal (4053, Cell Signaling; 5,000 x dilution), and anti-PKM1 rabbit monoclonal (7067S, Cell Signaling; 5,000 x dilution). Polyclonal antibodies against ADO (ab198245, abcam; 5,000 x dilution) and CDO-1 (ab53436, abcam; 5,000 x dilution) were used for Western blotting. Polyclonal antibodies against ADO and CDO-1 were purchased from Sigma to conduct Western blotting and immunohistochemistry [12, 62, 64]. Uncropped data of Western blotting analyses were shown in **Supplementary Figures 16-1, -2, -3 and -4.** For immunohistochemistry, tumour-bearing liver tissues were sectioned with 5-μm thickness, and

were fixed on the slide glass for 20 min with 4% (w/v) paraformaldehyde. Immunohistochemistry was performed using anti-CDO rabbit polyclonal antibody (dilution rate; 100 x for CDO) for 16 hours with blocking solution. After extensive washing with PBS, slides were incubated with the secondary antibody (Anti-rabbit IgG-HRP conjugated, W4011, Promega; 250 x dilution). Immunoreactivities were visualized using DAB substrate kit (SK-4100, Vector Laboratories, Burlingame, CA) according to manufacturer’s manuals. Following visualization, slides were counterstained with hematoxylin (#30011, Muto pure chemicals, Tokyo, Japan).

Measurements of retinol palmitate, retinol and beta-carotene

The mouse liver tissues were snap frozen in liquid nitrogen, then powdered by metal corn homogenizer (MBG-101C, Yasui Kikai, Osaka, Japan). Liver homogenate were filled up to 1ml with H_2O, then applied to Isolute SLE+ column (Biotage, Uppsala sweden), followed by elution with 5 ml dichloromethane according to supplier’s protocols. The organic extracts were evaporated to dryness under nitrogen stream. The residues were dissolved in 10 µl of pyridine and 50µl of the reagent BSTFA+TMCS (99:1) (#33148 Supelco, PA, USA) for trimethylsilylation. Retinol palmitate was detected by GC-MS. Retinol palmitate (46959-U, Supelco), and beta-carotene (C0560, Tokyo Chemical Industry) were used as standard. GC-MS analysis was performed on a Shimadzu GC-MS QP2010 Ultra equipped with an AOC20i autoinjector, and Rtx-5MS column (30m, 0.25 mm inner diameter, 0.25µm fd) in the 70eV electron ionization mode. The oven temperature program was as follows: 200ºC for 1 min, 20ºC/min to 320ºC, where the temperature was kept for 10 minutes. The carrier gas was helium with a constant flow speed of 39.0 cm/sec. Two ml was injected in 2:1 split ratio with an injector temperature of 250ºC, MS interface temperature was held at 280ºC. Selected ion monitoring for quantification was performed by recording the ions at *m/z* 284.20 for retinol palmitate, and *m/z* 536.45 for beta-carotene.

LC-MS measurements of sulfur-containing metabolites

GSH, GSSG, hypotaurine (HT) and taurine in frozen tissues were measured using Nexera UHPLC system coupled with LCMS-8030plus triple quadrupole mass spectrometer (Shimadzu, Kyoto Japan) according to previous methods (**Supplementary Note**) [12, 62, 63, 64]. A BEH Amide column (ACQUITY UPLC BEH Amide, 1.7 µm, 2.1 × 150 mm; Waters) was used for the separation of the metabolites. The mobile phase consisted of water containing 0.1% formic acid (A) and acetonitrile containing 0.1% formic acid (B). A gradient program was used as follows: 0-1min, 95% B; 1-3 min, 77% B; 3-11 min, 75% B; 11-14 min, 25% B; 14-20 min, 15% B; 20-21 min, 95% B; and held for 4 min. The injection volume was 2 µl. Column oven

was kept at 40°C and the flow rate was set to 0.2 ml min^{-1}. The multiple reaction monitoring (MRM) transitions were *m/z* 124.00>79.80 for taurine, *m/z* 611.25>306.1 for GSSG, *m/z* 194.05>79.90 for 2-morpholino-ethanesulfonic acid (an internal standard) in negative mode, *m/z* 109.85>30.10 for HT, *m/z* 308.10>179.05 for GSH in positive mode. The interface ionization potential was set at 4.5 kV with a temperature at 400°C. The flow rates of nebulizer and drying gases were set at 1.5 and 10 l min^{-1}, respectively. Contents of GSH, GSSG, HT and taurine in frozen brain samples were 4.0 ± 0.2, 1.2 ± 0.1, 0.13 ± 0.02 and 55.3 ± 6.2 (μmol g^{-1}, mean ± SE of 6 separate experiments), respectively. These results indicated that the ratios of GSH/GSSG and HT/taurine were 3.6 ± 0.3 and 0.003 ± 0.0001, respectively.

Determining contents of polysulfides in tumour-bearing brain

We determined contents of endogenous polysulfides by LC-MS using the monobromo-bimane-induced derivatization method [39, 49]. Using this method, tissue contents of polysulfides were compared between the tumour-free control and tumour-bearing brain tissues (TF and TB in **Supplementary Figure 15e**, respectively), which were deep frozen by liquid nitrogen. To this end, the block of the frozen brain was sliced first with multiple slices with 10-μm thickness with cryostat (CM3050, Leica) under epi-illumination to recognize GFP fluorescence, which featured tumour regions. After confirming the presence of the GFP-positive resection stamp, a 2-mm tissue slice was cut and trimmed to eliminate the GFP-negative surrounding tissues carefully by the surgical blade. The tumour-containing tissues which were prepared according to these procedures usually contained the surrounding tissues with 1.0~1.5 mm widths, and were defined as tumour-bearing tissues (TB in Suppl. Fig. 15e). As the tumour-free (TF) controls, the right hemispheres were collected from the frozen brain samples.

The brain tissue (~200 mg per a sample) was placed in two volumes of ice-cold 5 mmol l^{-1} monobromobimane (Invitrogen) in 10 mmol l^{-1} Tris-HCl (pH7.5) in 2 ml polypropylene tubes, homogenized at 4 °C with 3.0-mm zirconium beads using MicroSmash (Tomy Seiko), and then incubated for 10 min on ice. Methanol (10% vol/vol) was added to precipitate proteins. The mixture was vortexed and then centrifuged for 15 min at 20,400 × *g*. The supernatant was desalted through a column containing polymeric water-wettable reversed phase sorbent (Oasis HLB cartridge, WAT200677; Waters), and filtered through a centrifugal filter (Millipore Ultra free-MC; 5-kDa cutoff). Aliquots of 10 μl were analyzed to quantify sulfide dibimane (SDB) through liquid chromatography-electrospray ionization-tandem mass spectroscopy (LC/ESI/MS/MS). This method has been thought to provide overall contents of H_2S [37], but recent reports by Akaike, et al. [49] provided evidence for SDB generation results from not only H_2S but also organic and inorganic polysulfides, and thus allowed us to examine contents of the endogenous polysulfides in frozen brain samples without the addition of excess substrates for

H_2S-generating enzyme systems (e.g. CBS and CSE).

Analyses of cell viability and oxidative stress

Using several different human-derived cell lines described in the previous section, cell viability was assessed by CellTOX Green Cytotoxicity Assay kit (Promega, Madison, WI) according to the manufacturer's instructions. In brief, 1 x 10^4 cells were seeded in 96-well plate, and the cells were incubated in the medium with 1% or 10% FBS. The cells were treated with 2-deoxy-glucose (D8375, Sigma) for 16 hrs. The cells were incubated with CellTOX Reagent for 30 min. Following the washes with PBS three times, the fluorescence intensity (excitation/emission = 485nm/520nm) of CellTOX Green was detected using SpectraMAX M2 microplate reader (Molecular Devices, Sunnyvale, CA). The values of cell death were normalized by the fluorescence intensity of counterstained Hoechst dye (excitation/emission = 355 nm/465 nm). Intracellular levels of reactive oxygen species (ROS) were determined with CellROX Orange (C10443, Invitrogen), the fluorescent probe to measure oxidative stress in live cells according to manufacturer's manuals. In these experiments, MCF7, MDA-MB-231, PC9 and H1650 were examined to measure ROS (**Supplementary Figure 14b**), while we were unable to measure it in a reliable manner in H2170 because of a paucity of cell proliferation. In brief, 1 x 10^4 cells were seeded in 96-well plate, and then the cells were treated with sulfasalazine (SSZ, S0580, Tokyo Chemical Industry, Tokyo, Japan) for 24 hrs in DMEM containing with 1% FBS. After treating with SSZ, the cells were incubated in the medium containing 5 mmol l^{-1} CellROX Orange reagent and NucBlue Live Cell Stain ReadyProbes Reagent for 30 min at 37°C. Following the washes with PBS three times, intracellular ROS levels were measured with SpectraMAX M2 microplate reader (excitation/emission = 545 nm/565 nm). Endogenous ROS levels were normalized by the fluorescence intensity of Hoechst dye. To assess the efficiency of HT against oxidative stress, the cells were pretreated with 10 mmol l^{-1} 2-DG in DMEM with 1% FBS for 24 hrs, and followed by administering 5 mmol l^{-1} CellROX probe for 30 min, and then 0.25 ~ 1.0 mmol l^{-1} HT for 4 hrs for HCT116 cells (**Supplementary Figure 13**). After washing with PBS three times, intracellular ROS levels were measured to be compared among groups.

Supplementary Figures

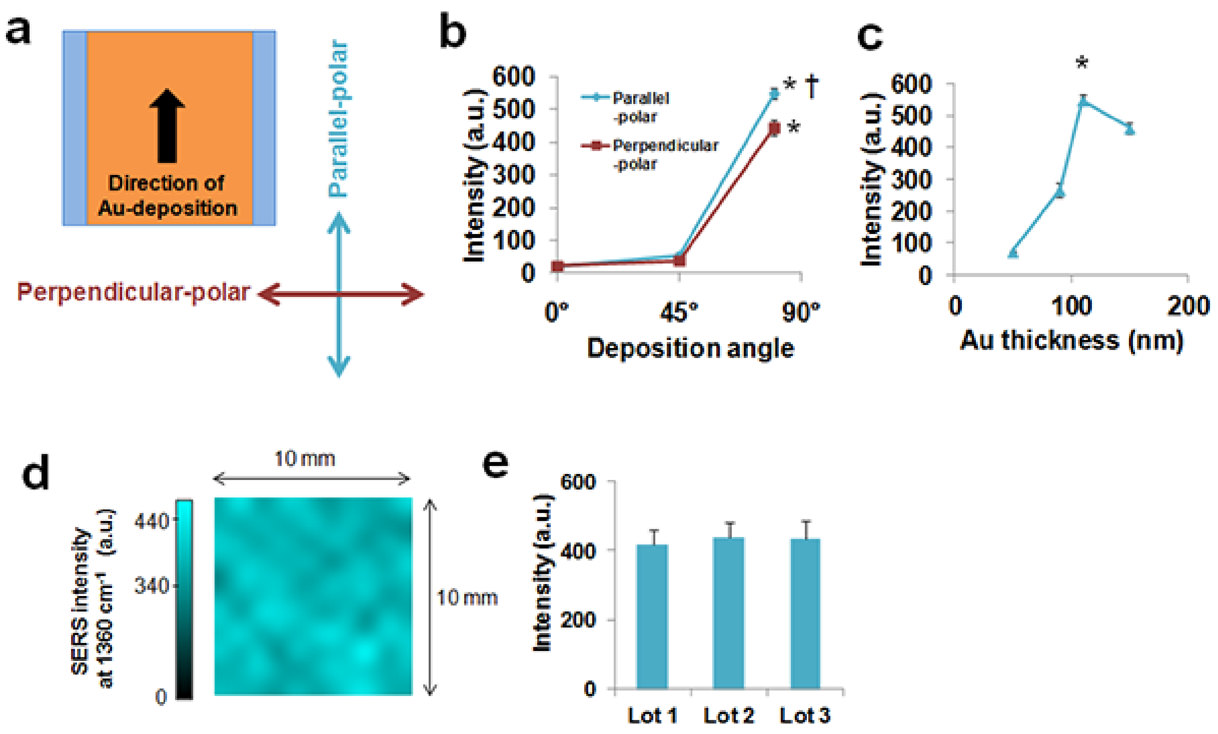

Supplementary Figure 1 Characterization of gold-nanofève (GNF) substrate. (**a**) Definition of polarized light. Parallel- and Perpendicular-polar indicate the parallel and side directions of polarized light, respectively. (**b**) Alterations in intensities of SERS signals derived from rhodamine 6G (R6G) by different deposition angles. In this experiment, 40 μL of R6G solution (100 μmol l^{-1}) was deposited on the GNF substrates and dried in air. Data were 3 separate experiments under the condition using aluminum films with 50 nm in thickness. *$p < 0.05$ versus the data determined at 0° and 45° in the Au deposition angles. † $p < 0.05$ versus the S-polar data measured at 80° in the Au deposition angle. ANOVA with Fischer's LSD test ($F_{(5,12)}$ =292.546). (**c**) Dependence of Au thickness on sensitivity of SERS substrate. The deposition angle of Au was 80° and the Au thicknesses were measured at the deposition angle of 0°. *$p < 0.05$ versus the data determined at 50, 90, 150 nm of Au thickness. ANOVA with Fischer's LSD test was used for analyzing statistical significance ($F_{(3,8)}$ =125.515). (**d**) A representative image showing spatial uniformity of SERS signals of R6G determined at 1360 cm^{-1}. As described in the main text, the coefficient of variation is 11.0% ± 0.7% (n=3); the value was comparable to that in GNC substrates which we reported previously [15]. (**e**) Comparison of the R6G sensitivity among 3 different lots of GNF substrates. SERS signal intensities are comparable among different lots. In these experiments, GNF substrates were immersed into an R6G solution (10 μmol l^{-1}) for 15 min, rinsed lightly by pure water, and dried in air.

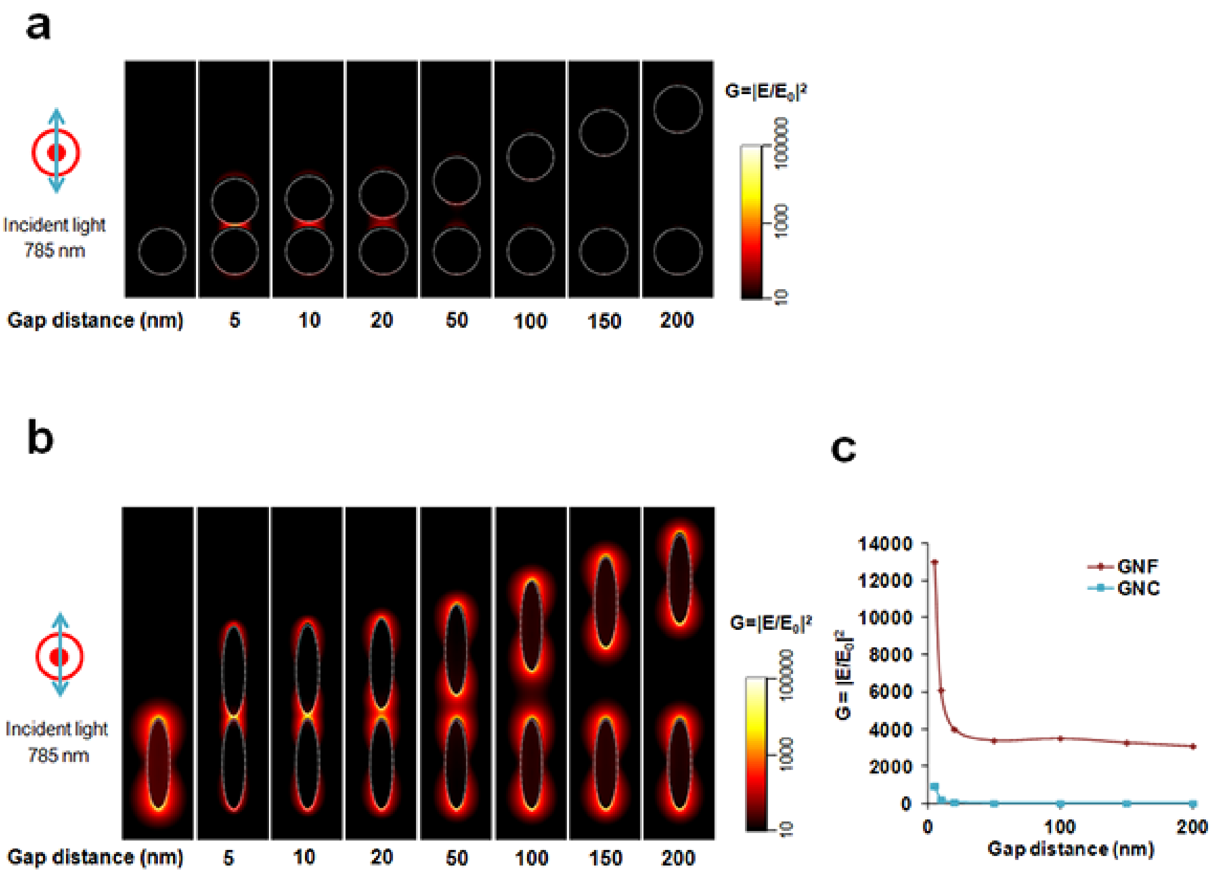

Supplementary Figure 2 Differences in the local field enhancement (G) between GNC **(a)** and GNF **(b)** calculated by FDTD simulation. Heat maps indicate magnitudes of the enhancement. **(c)** G values calculated at 1 nm distant from the apex of the Au-nanoparticle, and these values were plotted as a function of the gap distance between the two adjacent Au-nanoparticles. Note that the local field enhancement of GNF, but not of GNC, is maintained without regression.

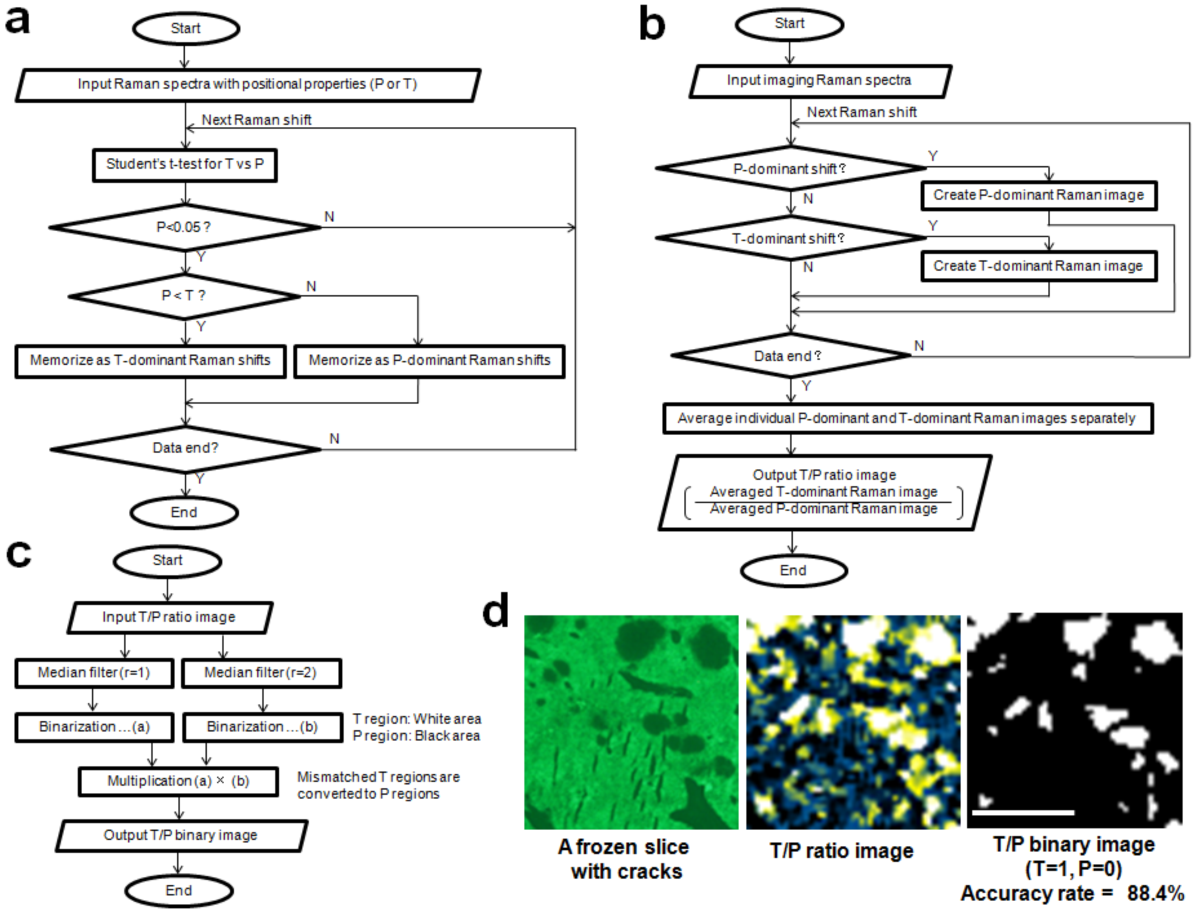

Supplementary Figure 3 Flowcharts for extracting tumour boundaries in tumour-bearing liver tissues. (**a**) Selection of tumour (T)- and parenchyma (P)-dominant Raman shifts from shControl-xenografted liver tissues for building parent database for automated extraction of tumour boundaries. (**b**) Flowchart to establish accumulative lists of Raman shifts (ALR in **Fig. 4**) to compose the ratio images between T-dominant and P-dominant Raman images. T-bar and P-bar indicate average values of T- and P-dominant Raman shifts. (**c**) Schematic diagram of triangle auto-thresholding method to extract the boundaries between tumours and parenchyma (r=1: Median filter by 1 pixel; r=2: Median filter by 2 pixels). (**d**) A representative picture indicating disappearance of cracks on frozen tissue sections through triangle thresholding method. The sample was extracted from the shControl group. Interestingly, we serendipitously observed that cracks on frozen tissue sections were automatically excluded from signals in the final T/P binary image - an advantage of this image-processing method for identifying tumour boundaries. In this representative case, the accuracy rate of the T/P binary image was 88.4%. Scale bar = 1 mm.

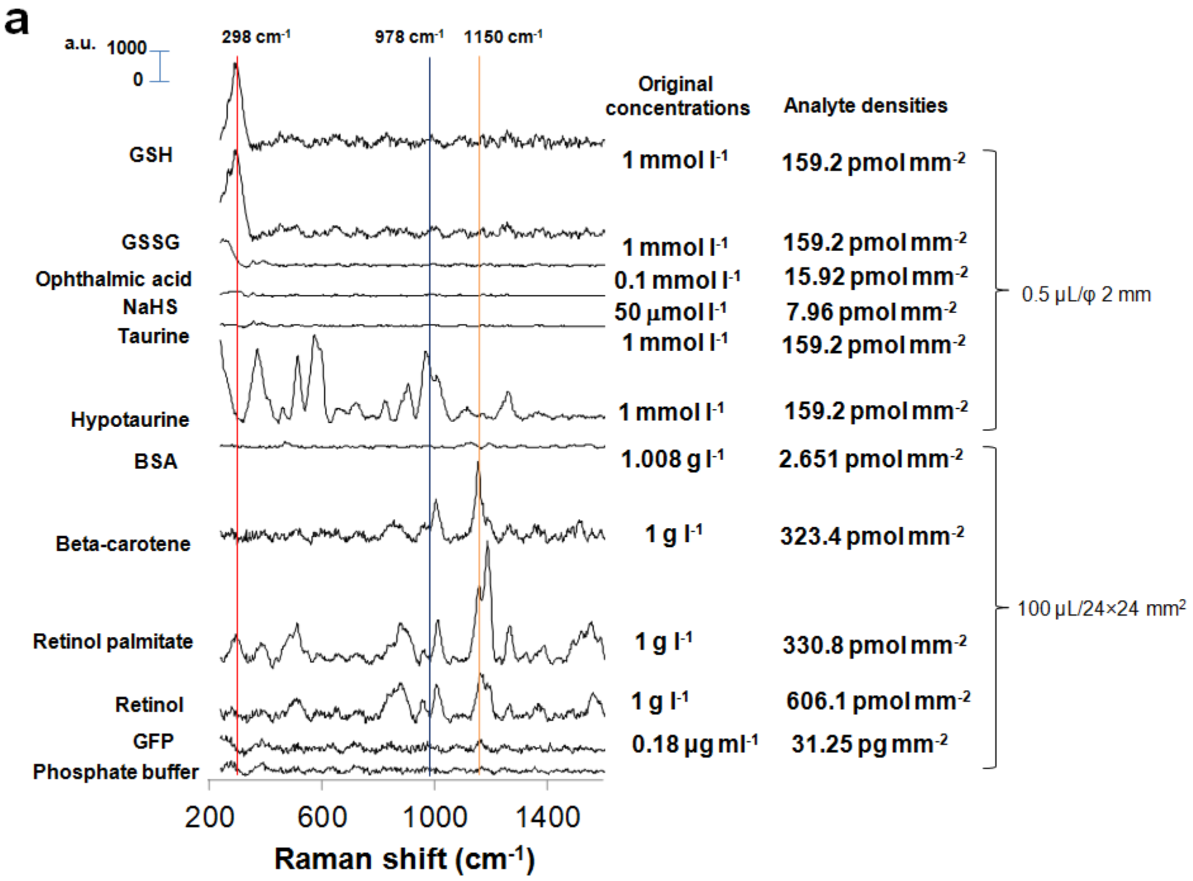

Supplementary Figure 4 (a) SERS spectra of GSH, GSSG, ophthalmic acid, NaHS, taurine and hypotaurine. Note that ophthalmic acid, in which cysteine residue of GSH was replaced with 2-aminobutylate, generated no detectable shift at the same wave number, indicating that cysteine residue of GSH is responsible for binding to Au to enhance the Raman shift at 298 cm^{-1}. Among these analytes, hypotaurine exhibits a robust SERS signal at 978 cm^{-1} that was not detected in other analytes. Bovine serum albumin (BSA) that has a reactive thiol in its structure does not exhibit robust SERS peaks between 200 cm^{-1} and 1600 cm^{-1}, suggesting little involvement of protein thiols in SERS enhancement, if any. Beta-carotene, retinol palmitate and retinol display robust peaks at 1150 cm^{-1}. GFP at the given analyte density did not yield specific GNF-SERS signals under the current experimental conditions. Phosphate buffer at 0.1 mol l^{-1} was dried on the GNF substrate, and was used as a GFP-free control solution.

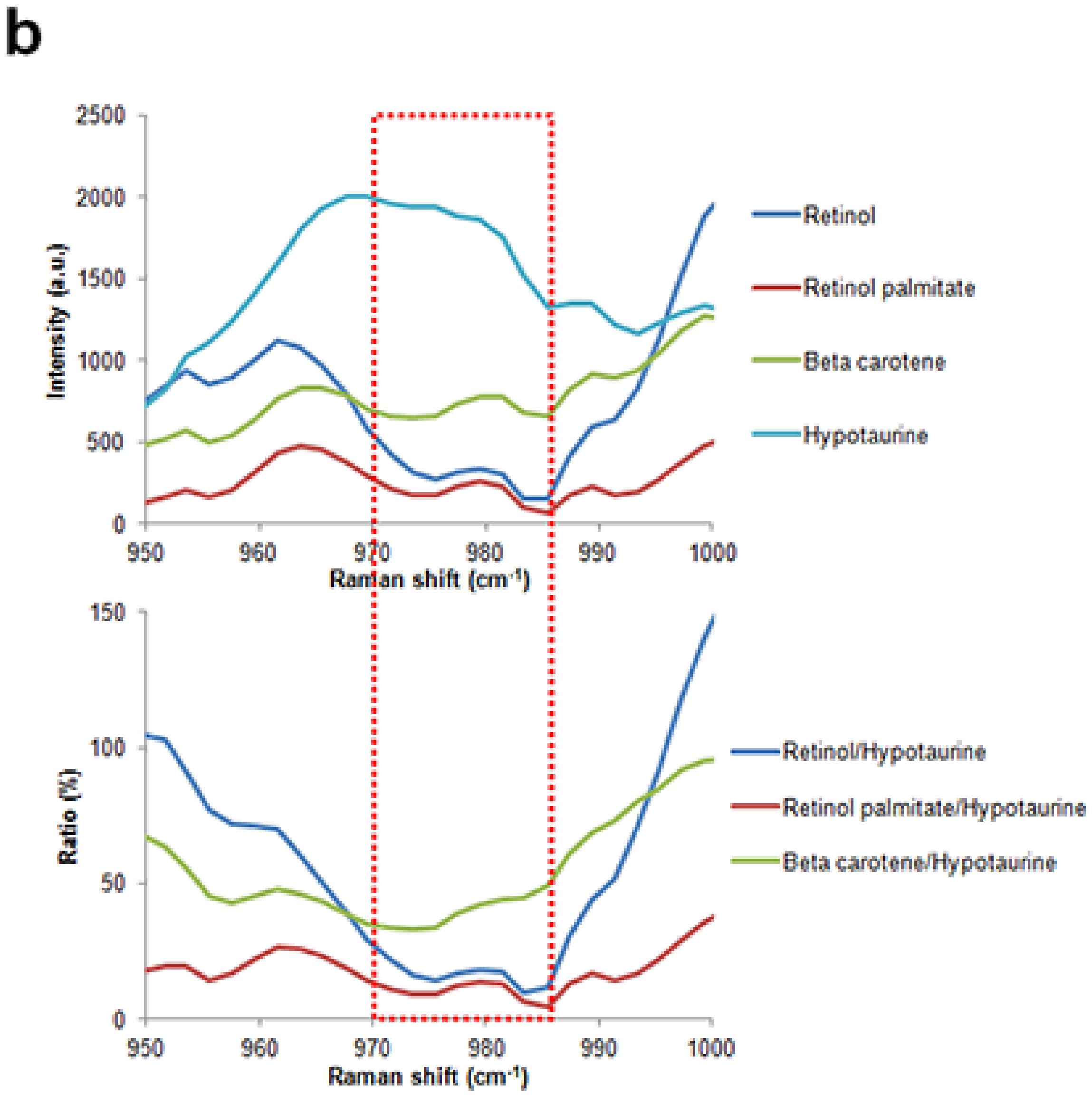

Supplementary Figure 4 (Continued) (b) Differences in SERS spectra of hypotaurine and retinol palmitate in vitro. Hypotaurine and retinol palmitate were dissolved in methanol at 50 μmol l^{-1}. The SERS peak derived from beta-carotene might interfere with measurements of hypotaurine, but the contents of beta-carotene were undetectable in vivo under the given experimental conditions (See Page 2 of **Suppl. Info**). Evaluation of the ratio between the hypotaurine peak versus the retinol palmitate (Retinol palmitate/Hypotaurine) peak or the retinol peak (Retinol/Hypotaurine) were minimum in a range between 970 cm^{-1} and 986 cm^{-1}, and thus the optimal wave range to exclude RP and R was 978 ± 10 cm^{-1} to determine hypotaurine.

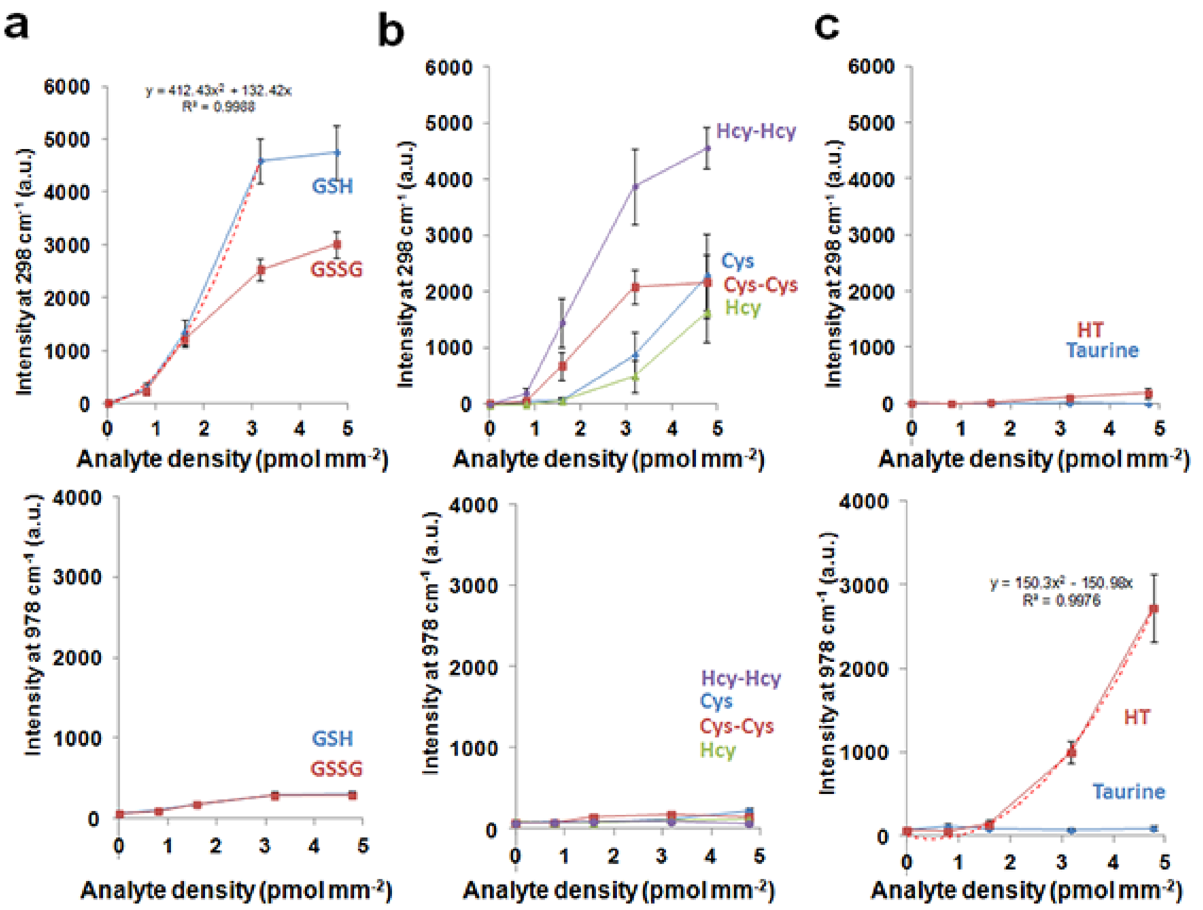

Supplementary Figure 5 Calibration of SERS signal intensities as a function of analyte densities. (**a**) The dose-response curves of GSH and GSSG. The SERS signal of GSH reached a plateau level at the analyte density of 5 pmol mm^{-2}, suggesting saturation of the analyte on GNF substrates, while that of GSSG occurred at the same wave number at 298 cm$^{-1,}$ and was weaker than GSH. Both analytes do not show any notable SERS signals at 978 cm^{-1}. (**b**) The dose-response curves of SERS signals derived from homocystine (Hcy-Hcy), homocysteine (Hcy), cystine (Cys-Cys) and cysteine (Cys) at 298 cm^{-1} and 978 cm^{-1}. (**c**) The dose-response curves of SERS signals derived from hypotaurine (HT) and taurine. To calibrate the SERS signal in the tumour-bearing liver tissues at 298 cm^{-1} and 978 cm^{-1}, quadratic curve fitting was carried out; the curve for GSH at 298 cm^{-1} is expressed as $Y=412.43\ X^2\ +132.42X$ ($R^2 = 0.99$), while that for HT at 978 cm^{-1} is $Y=150.3X^2-150.98X$ ($R^2=0.998$). These results indicate that the detection limit of HT is approximately 1.0 pmol mm^{-2}.

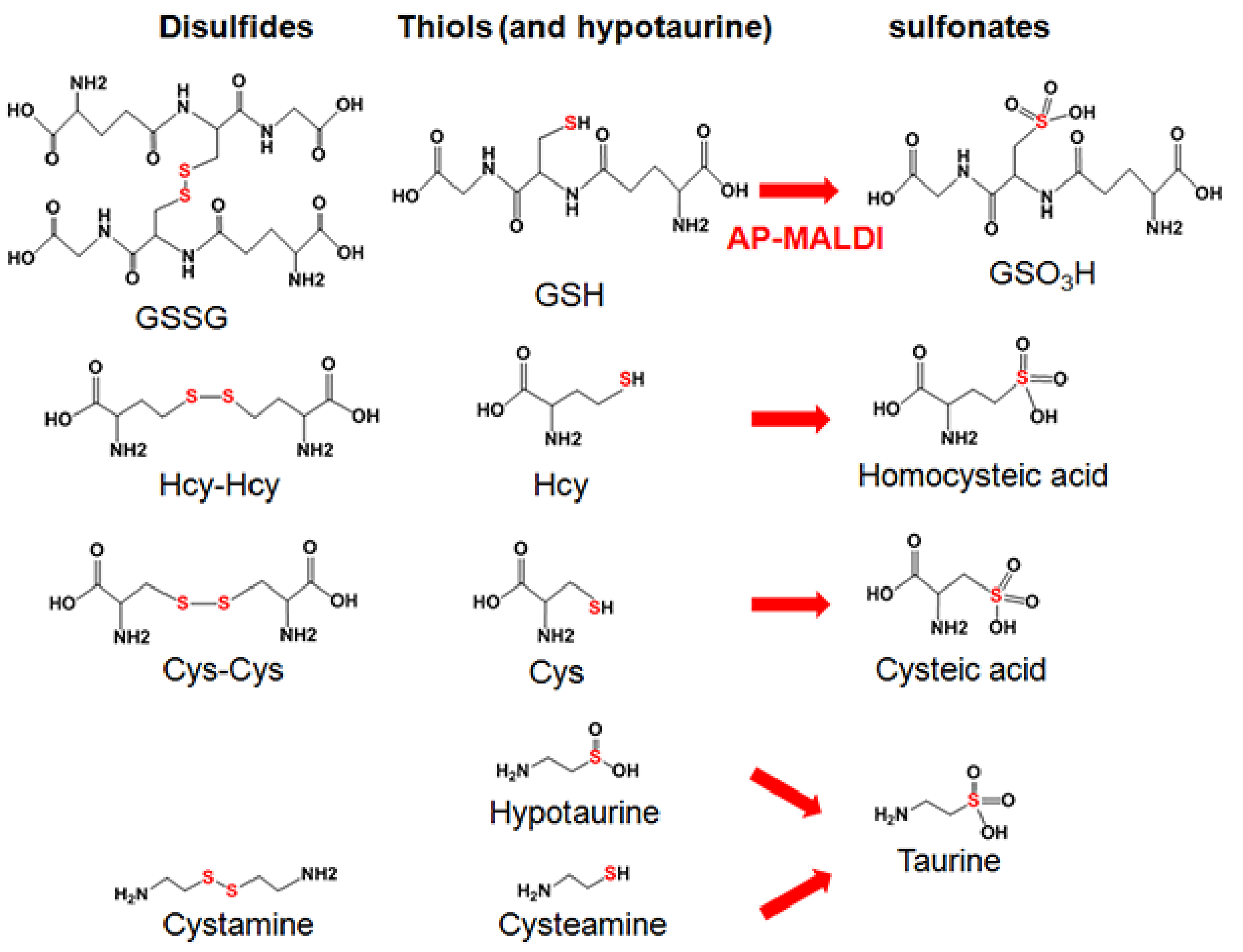

Supplementary Figure 6 **Putative diagram of MALDI-induced photo-oxidation of GSH, Hcy, Cys, hypotaurine and cysteamine.** Through being exposed to MALDI, GSH, Hcy and Cys are converted to glutathione sulfonate, homocysteic acid and cysteic acid, while hypotaurine and cysteamine are converted to taurine. AP-MALDI: Matrix-assisted laser desorption and ionization under the atmospheric pressure.

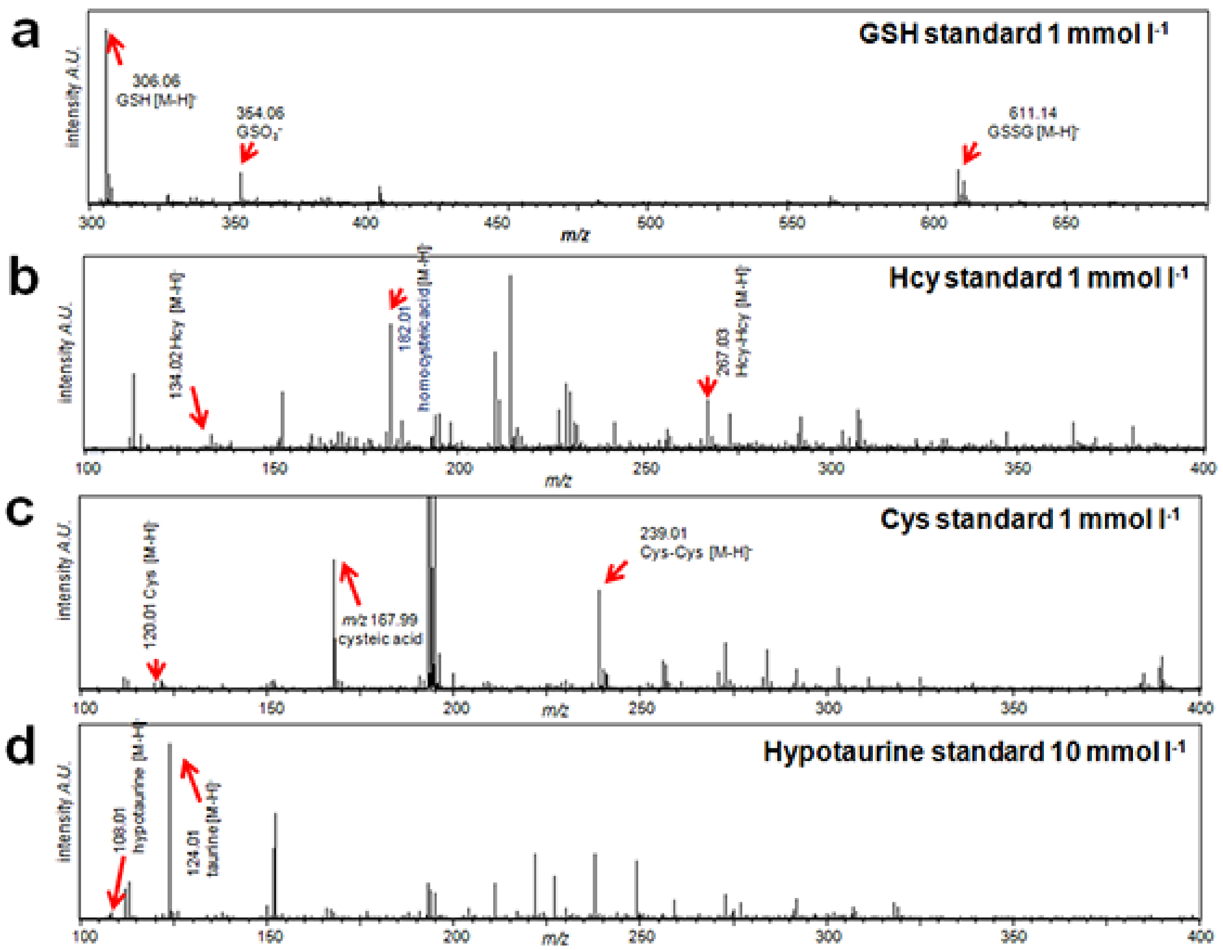

Supplementary Figure 7 Oxidation of standard thiols and hypotaurine under atmospheric pressure MALDI conditions. (**a**) Conversion of the standard GSH to glutathione sulfonate (GSO_3^-) and oxidized glutathione (GSSG) under atmospheric pressure (AP)-MALDI conditions. (**b**) Conversion of homocysteine (Hcy) to homocystine (Hcy-Hcy) and homocysteic acid. (**c**) Conversion of cysteine (Cys) to cystine (Cys-Cys) and cysteic acid. (**d**) Conversion of hypotaurine to taurine. Hypotaurine is highly prone to MALDI-induced oxidation and serves as a source of taurine as the terminal sulfonated product (*m/z* 124.01).

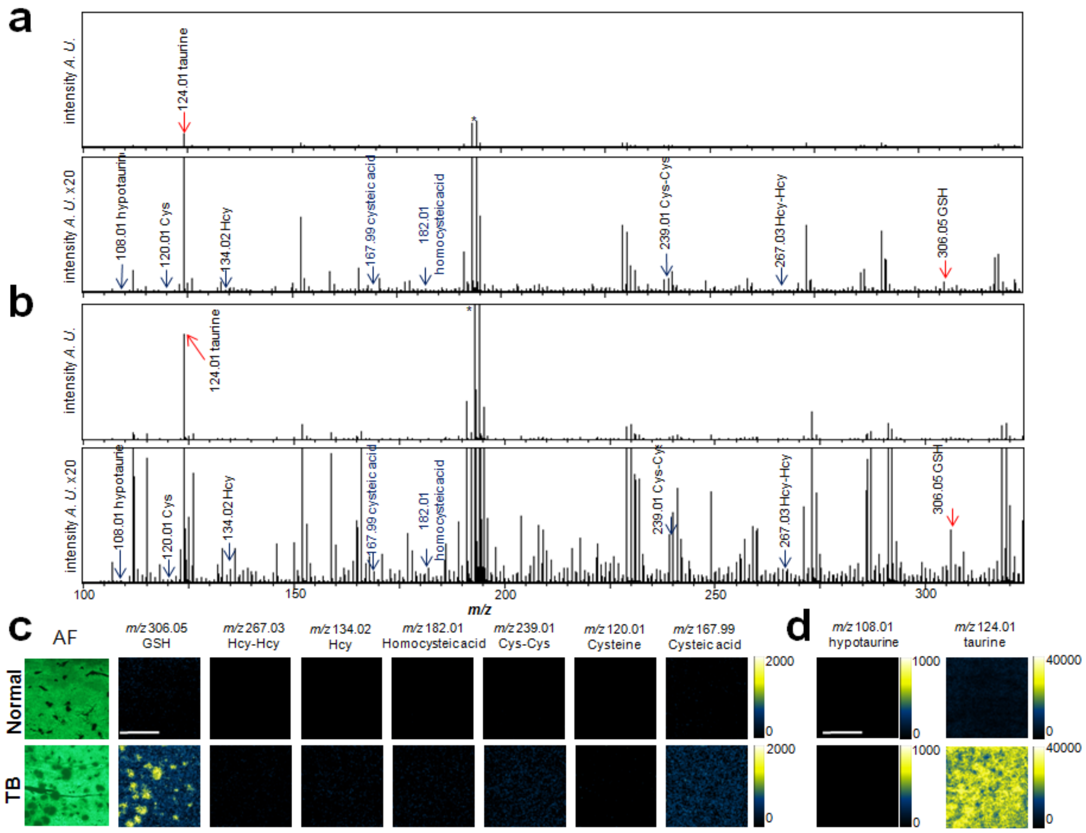

Supplementary Figure 8 Imaging mass spectrometry of the normal and tumour-bearing livers in a range between *m/z* 100 and *m/z* 325. (**a**) A representative spectrum of the normal liver (one of 5 separate experiments), showing that taurine constitutes the largest peak at *m/z* 124.01. The lower panel indicates the identical spectrum in which Y-axis is magnified by 20 folds. Red and blue arrows depict positive and negative mass peaks, respectively. (**b**) A representative spectrum of the tumour-bearing liver (one of 7 separate experiments), again showing that taurine constitutes the largest peak at *m/z* 124.01. The lower panel indicates the identical spectrum in which Y-axis is magnified by 20 folds. Red and blue arrows depict positive and negative mass peaks, respectively. To determine specific mass peaks of Hcy-Hcy, Hcy, Cys-Cys and Cys, and their sulfonated end products homocysteic acid and cysteic acid, the *m/z* values of their specific mass fragments were gated within a range of *m/z* ± 0.01 to minimize noise signals surrounding the specific peaks of the compounds. (**c**) Imaging mass spectrometry in the normal and tumour-bearing (TB) livers. Hcy-Hcy, Hcy, homocysteic acid, Cys-Cys, Cys, and cystic acid exhibited few signals in SERS imaging, if any, while, under the same conditions, GSH imaging in the same microscopic field showed that the mass signal occurs predominantly in tumours rather than in parenchyma. (**d**) Imaging MS of hypotaurine and taurine in the control and tumour-bearing (TB) livers. The data were collected from the same microscopic field as Panel (c). Scale bars = 1.0 mm. All scales indicate arbitrary units (a.u.).

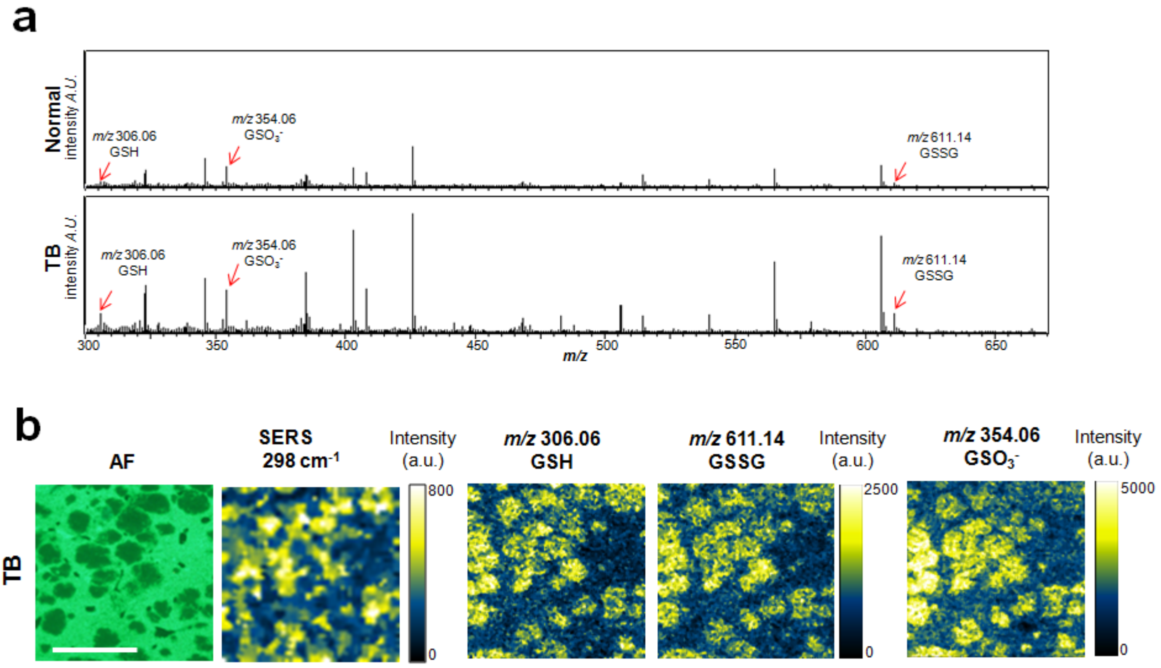

Supplementary Figure 9 Comparison of SERS images at 298 cm^{-1} and those of GSH, GSSG and glutathione sulfonate (GSO_3^-). (**a**) Representative SERS images and imaging MS for GSH, GSSH and GSO_3^- in the tumour-free normal livers and tumour-bearing (TB) livers of NOG mice. C and T indicate central veins and tumours, respectively. The autofluorescence (AF) image and SERS image at 298 cm-1 were collected from the identical tissue section, while imaging MS pictures for GSH, GSSG and GSO_3^- were collected from a serial section. Scale bar = 1.0 mm. Note that notable SERS signals in central veins results from glutathione in erythrocytes contained in the tissue section. (**b**) Original mass spectra in a range between *m/z* 300 and *m/z* 670 of the regions shown in (**a**) in the normal and TB livers, indicating peaks of GSH, GSO_3^- and GSSG.

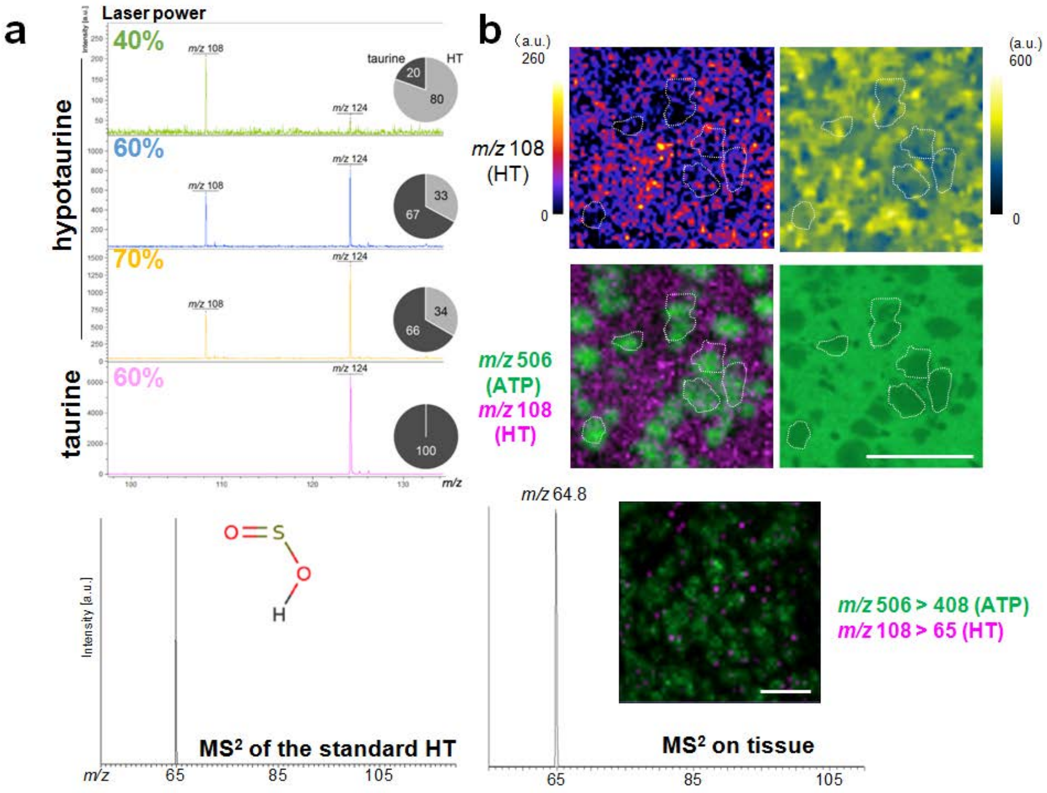

Supplementary Figure 10 **Photo-oxidative conversion of hypotaurine to taurine.**
(**a**) Photo-oxidation of the standard sample of hypotaurine (HT) to taurine under vacuum-type MALDI conditions. the standard HT sample generated secondarily oxidized taurine under lowest magnitude of laser irradiation for MALDI (40% of the maximum laser intensity). Note that the increasing laser power did not increase the mass peak of HT linearly, because a portion of HT was auto-oxidized into taurine. The pie charts indicated the percentage ratios of HT versus taurine for individual laser power conditions, suggesting that greater than half of HT was oxidized into taurine under the laser power of >60%. The bottom of Panel (a) indicated MS of the standard taurine. (**b**) A representative vacuum-type MALDI-MS imaging showing distribution of HT in the tumour-bearing livers. SERS imaging at 978 cm^{-1} in the serial tissue section exhibited a comparable geographic pattern of HT in tissues. Superimposition of mass signals of HT (*m/z* 108) on those of ATP (*m/z* 506) which marks tumours predominantly suggest that HT is present predominantly in parenchymal regions but not in tumours. Broken lines exhibit representative potions of metastatic tumours that indicated high ATP and low GNF-SERS at 978 cm^{-1}. Bar = 1.0 mm. (**c**) Tandem MS (MS^2) spectra collected from the standard HT sample (standard) and those from a tumour-bearing liver tissue (on tissue). Bar = 1.0 mm.

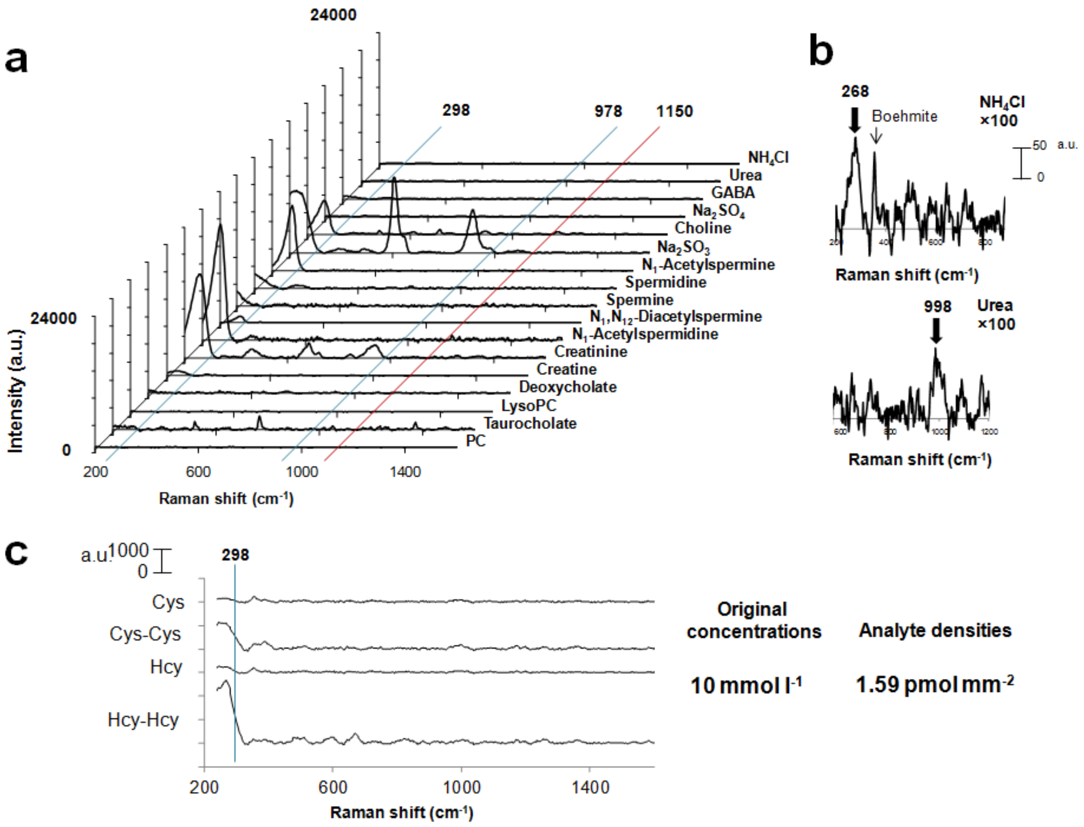

Supplementary Figure 11 SERS profiles of major small molecular metabolites in the mouse liver. (a) Note that the SERS signals of all metabolites examined in this figure are not overlapped with those at 298 cm^{-1}, 978 cm^{-1} and 1150 cm^{-1}. The relationship of the original concentrations of analytes in purified water to the analyte densities of individual compounds (pmol mm^{-2}) on GNF substrates are as follows: NH_4Cl, urea, Na_2SO_4, Na_2SO_3; 1 mmol l^{-1} (=159.2 pmol mm^{-2}), GABA, and choline; 100 mmol l^{-1} (=15.915 nmol mm^{-2}), N-acetyl-spermidine, spermidine, spermine, N_1,N_{12}-diacetyl- spermidine, N_1-acetylspermidine, creatinine, creatine, lyso- phosphatidylcholne (lyso-PC) and phosphatidyl choline (PC); 10 mmol l^{-1}, and deoxycholate and taurocholate; 20 mmol l^{-1} (= 3183.1 pmol mm^{-2}). Concentrations of lyso-PC, PC and deoxycholate and taurocholate are overall comparable to those in bile according to previous studies [26, 31]. (**b**) Data of SERS intensities for NH_4Cl and urea were magnified by 100 folds. The concentration of urea in these experiments is 0.113 mg l^{-1}. Considering that physiologic concentration of urea in circulation is approximately 0.1~0.2 g l^{-1}, the results suggest that the current SERS technology enables to detect urea in vivo at 998 cm^{-1}. (**c**) Representative spectra of homocystine (Hcy-Hcy), homocysteine (Hcy), cystine (Cys-Cys) and cysteine (Cys) at 10 μmol l^{-1} (= 1.59 pmol mm^{-2}). Note that physiological concentrations of these metabolites are estimated to be less than 10 μmol l^{-1} as judged from the data in **Table S2**.

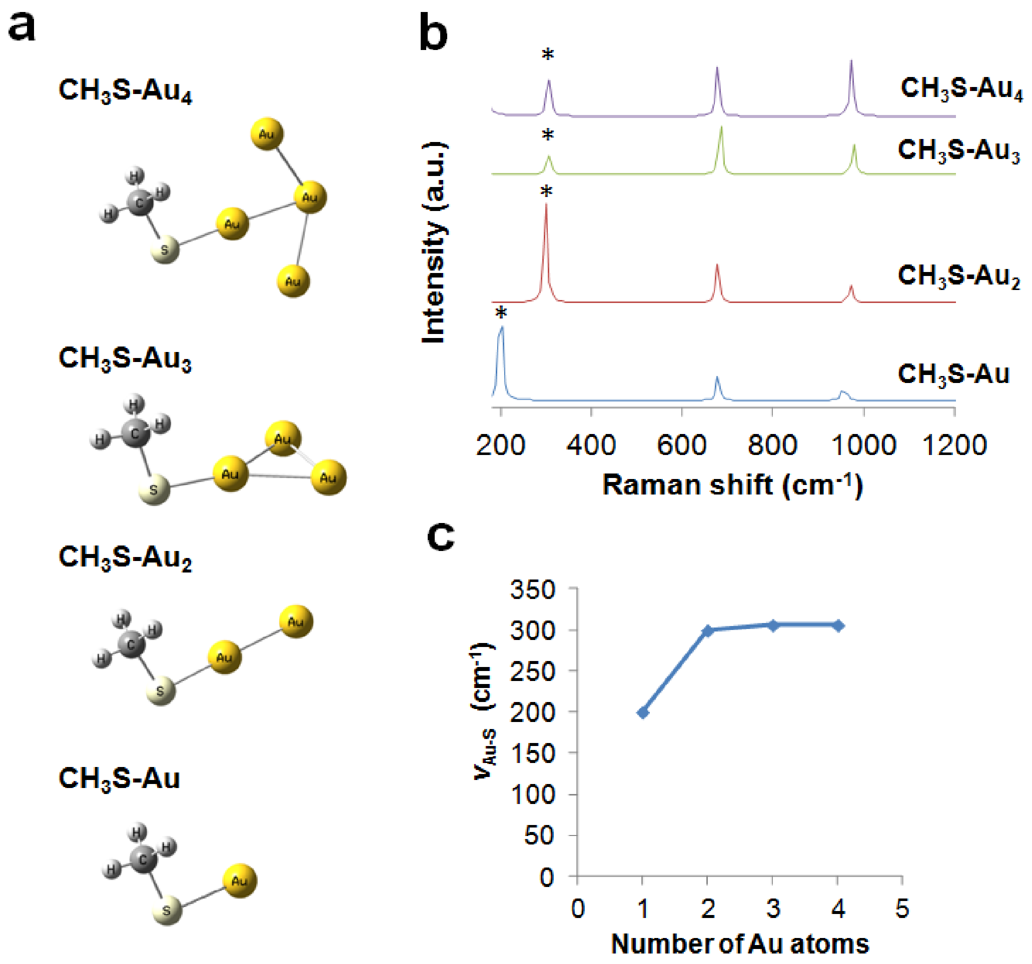

Supplementary Figure 12 Optimization of the structure of Au cluster for SERS spectra calculation using DFT method. (**a**) A model for calculating prediction of molecular vibrational modes of methyl mercaptane (CH_3S^-) binding to various numbers of Au atomic cluster. Molecular geometries were fully optimized at the CPCM-B3LYP/ 6-311+G*/SDD (Au) level, assuming that the molecules are located in H_2O. (**b**) Calculated Raman spectra of $CH_3S\text{-}Au_n$ (n=1~4). Peaks generated by the Au-S stretching mode were marked by asterisks. (**c**) Dependence between the frequency of Au-S stretching mode and the number of Au atoms for constructing their atomic clusters.

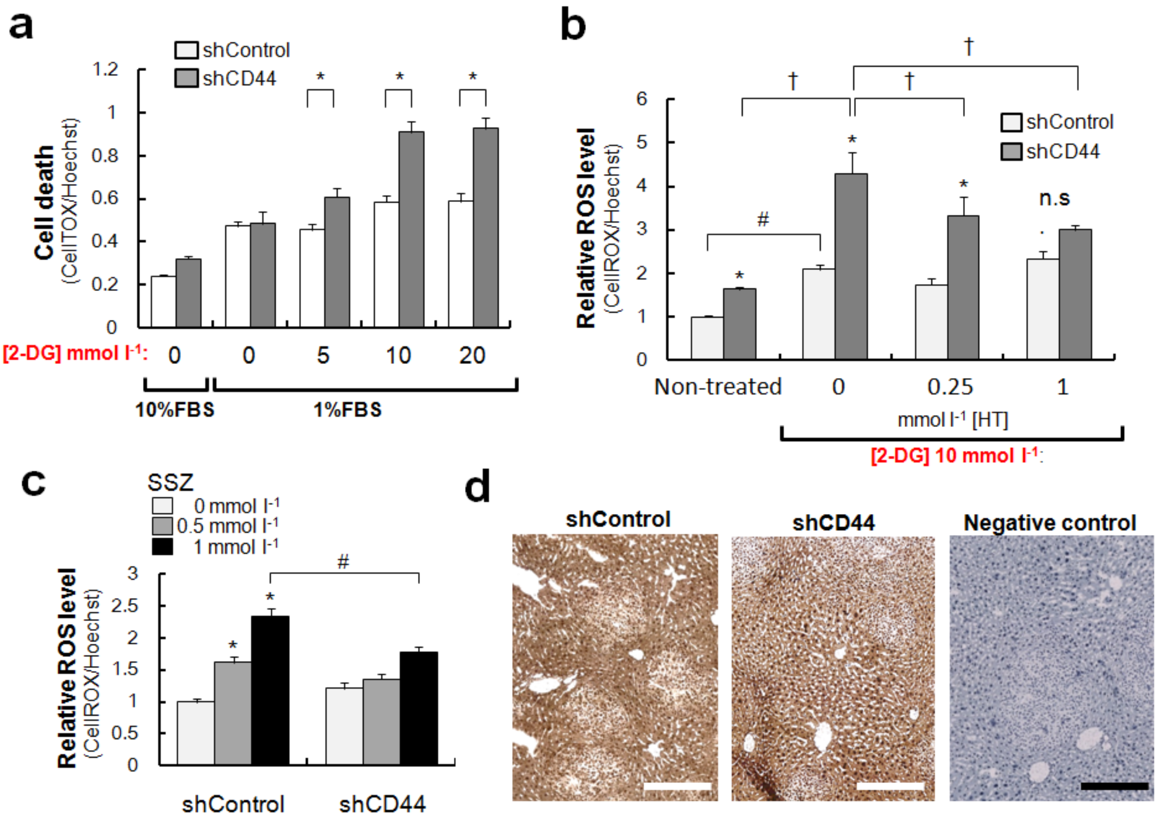

Supplementary Figure 13 **Enhanced cell death and oxidative stress in shCD44 cells.** (**a**) Different effects of 2-deoxy-glucose (2-DG) on cell death. Sensitivity to 2-DG treatment is significantly enhanced in shCD44 cells than in shControl cells. Data indicate mean ± SE of 6 separate experiments. *p< 0.05 tested by ANOVA with Fischer's LSD test. $F_{(9, 50)} = 36.434$. (**b**) Effects of exogenously administered HT on 2-DG-induced enhancement of oxidative stress. Data indicate mean ± SE of 5 separate experiments. *p < 0.05 versus shControl. # and † indicate p< 0.05 versus the groups treated without 2-DG. Differences were tested by ANOVA with Fischer's LSD test. $F_{(7, 32)} = 17.717$. (**c**) Effects of sulfasalazine (SSZ) on oxidative stress in shControl and shCD44 cells. Data indicate mean ± SE of 6 separate experiments. *p < 0.05 versus SSZ-free groups. #p< 0.05 analysed by ANOVA with Fischer's LSD test. $F_{(5, 30)} = 27.437$. (**d**) Immunohistochemistry of cysteine dioxygenase-1 (CDO-1) in tumour-bearing liver tissues. Note that liver parenchyma abundantly express, and tumours modestly express CDO-1. Bar = 500 µm

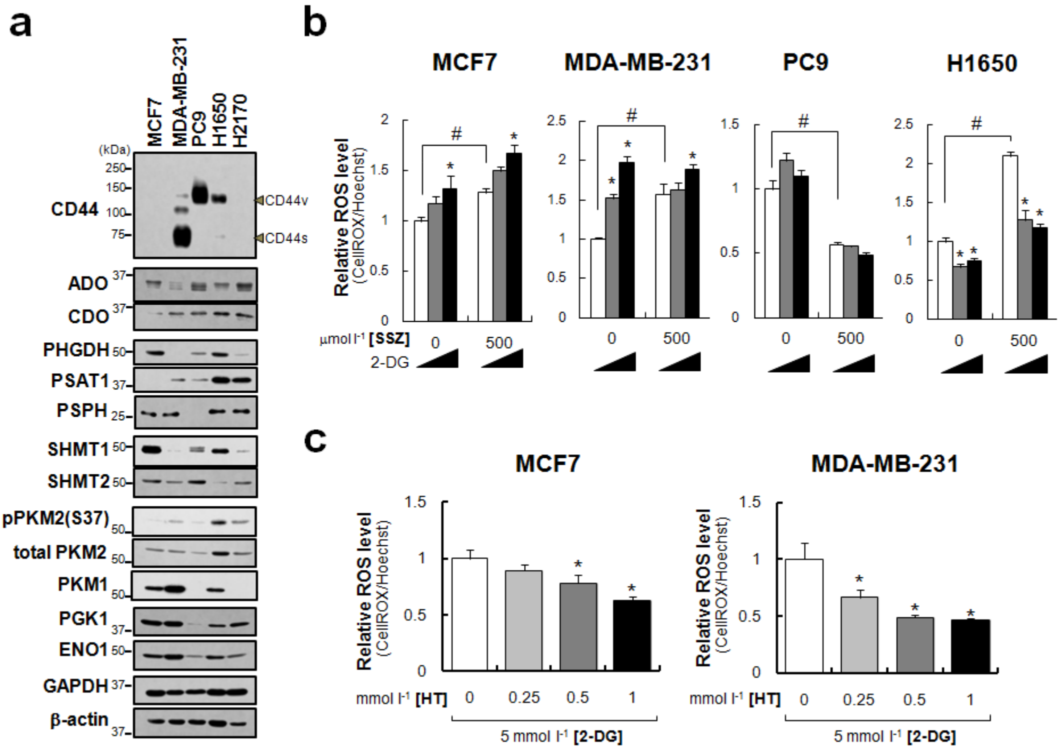

Supplementary Figure 14 Differences in responsiveness of reactive oxygen species (ROS) to sulfasalazine (SSZ) and 2-deoxy-glucose (2-DG) among a variety of human-derived cancer cell lines. (**a**) Western blot analyses of MCF7 and MDA-MB-231 cells (breast cancer cells), and PC9, H1650 and H2170 cells (non-small cell lung carcinoma cells that are resistant to SSZ). Among these cell lines, H2170 was not used for ROS assay because the cells exhibited very slow proliferation. Under cultured with 1%FBS, MDA-MB-231, PC9 and H1650 expressed considerable CD44 expression, while MCF7 displayed little CD44 expression, if any, suggesting that MCF7 serves as a CD44-low cell mimicking CD44 knockdown. All cell lines expressed both ADO and CDO, while expressing either PHGDH or PSAT1, the enzymes gating for serine/glycine cleavage systems to generate hypotaurine (HT). (**b**) Distinct ROS responses in MCF7 and MDA-MB-231 from those in PC9 and H1650 in the presence of SSZ (0 or 500 μmol l^{-1}) and 2-DG (0, 5 or 10 mmol l^{-1}). Note that SSZ at 500 μmol l^{-1} causes significant ROS increases in MCF7 and MDA-MB-231 that are further enhanced by 2-DG in dose-dependent manners; these events mimic SSZ- and 2-DG-induced ROS elevation in HCT116 cells treated with CD44 knockdown (**Suppl. Fig. 13b and 13c**). On the other hand, PC9 displayed SSZ-induced ROS suppression, and 2-DG-induced ROS elevation is undetectable. In addition, H1650 exhibited SSZ-induced ROS elevation, while showing significant ROS suppression by 2-DG. *$p < 0.05$ versus the group treated without 2-DG. $^{\#}p < 0.05$ versus the

groups treated without SSZ. Differences were tested by ANOVA with Fischer's LSD test. $F_{(5,24)}$=10.443 for MCF7, $F_{(5,30)}$=16.679 for MDA-MB-231, $F_{(5,29)}$=64.056 for PC9, and $F_{(5,30)}$=57.589 for H1650. Based on these data, we examined whether MCF7 and MDA-MB-231 display responsiveness of 2-DG-induced ROS elevation to hypotaurine (HT) application. (**c**) Inhibitory effects of exogenously applied HT (0, 0.25, 0.5, 1.0 mmol l^{-1}) on 2-DG-induced ROS elevation in MCF7 and MDA-MB-231 cells. Data indicate mean ± SE of 6 separate experiments. *$p< 0.05$ tested by ANOVA with Fischer's LSD test. $F_{(3, 20)} = 6.21$ for MCF7, and $F_{(3, 20)} = 8.972$ for MDA-MB-231.

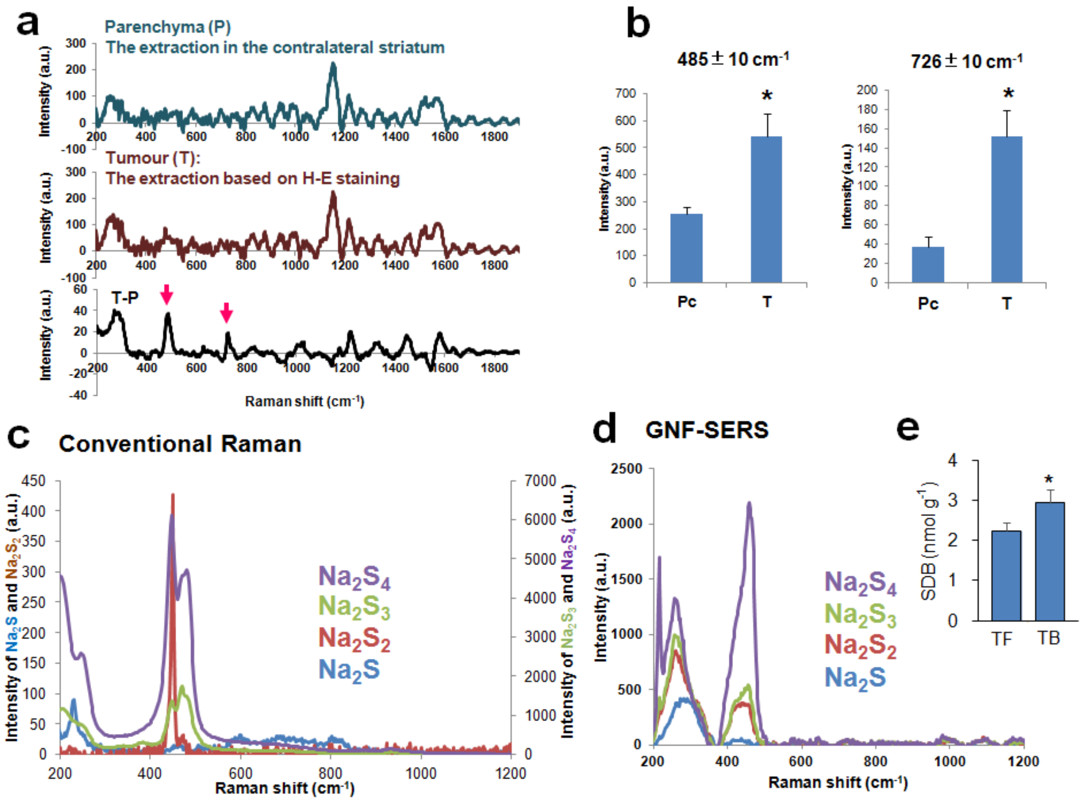

Supplementary Figure 15 Tumour-specific GNF-SERS spectral profiles in a syngeneic glioblastoma model (**a**) SERS spectra of the contralateral striatum parenchyma (P) and tumour regions (T), and the difference spectra between P and T. Arrows: 2 major T-dominant peaks at 485 cm^{-1} and 726 cm^{-1}. (**b**) Differences in the peak intensities at 485 cm^{-1} and 726 cm^{-1} between tumour (T) and parenchymal (Pc) regions. These data were accumulative signal intensities in a range of ±10 cm^{-1} for the SERS imaging protocols. *$P < 0.05$ by unpaired Student's t-test. T values for statistical analyses are as follows: T(5) for the peak difference at 485 cm^{-1} = -2.963, and T(5) for the peak difference at 726 cm^{-1} = -3.610. (**c**) Spectral analyses of crystallized sodium polysulfides (Na_2S, Na_2S_2, Na_2S_3, Na_2S_4) by conventional Raman spectrometry. Na_2S_3 and Na_2S_4 displayed splitted peaks at around 480 cm^{-1}, suggesting the presence of central and terminal S-S stretching modes. Experiments were performed according to the previous method described in Janz, G.J. et al.[48] (**d**) GNF-SERS spectra of sodium polysulfide in distilled water. The concentration of these reagents was 100 μmol l^{-1}. (**e**) Tissue contents of H_2S/polysulfides measured by monobromobimane-derivatization method. SDB: sulfide dibimane. TF: Tumour-free brain tissues, and TB: Tumour-bearing brain tissues. Mean + SEM of 10 and 5 separate experiments for TF and TB, respectively. *$P < 0.05$ as compared with TF. T(13) = 1.918 by unpaired Student's t-test.

Suppl. Fig.16 Uncropped blot. data-1

(Fig.6a)

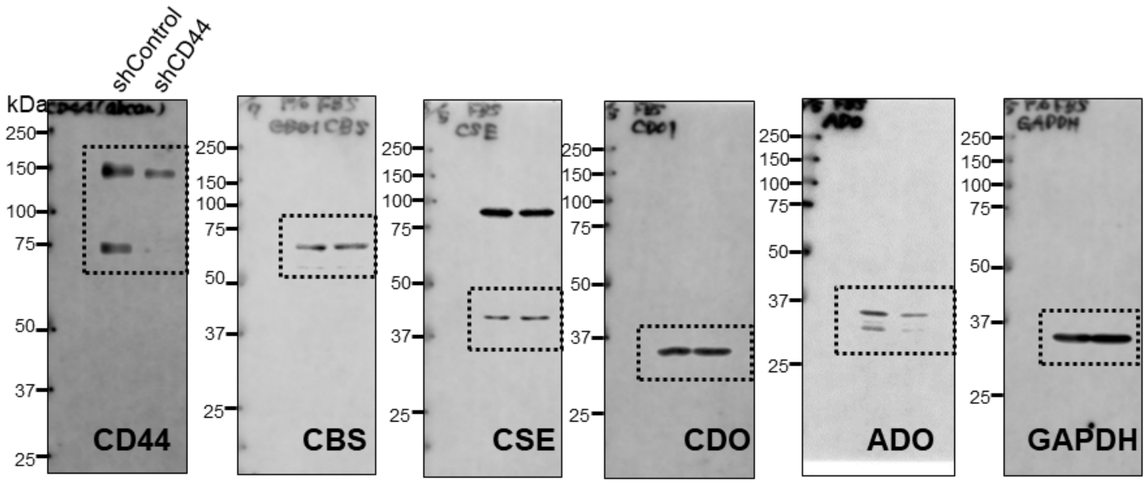

Supplementary Figure 16-1 Uncropped blotting data in **Figure 6a** showing comparison between shControl and shCD44 cells of CD44, CBS, CSE, CDO (CDO-1), ADO, and GAPDH. Dotted squares indicate partial images used in **Figure 6a**.

Suppl. Fig.16   Uncropped blot. data-2

(Fig.8d)

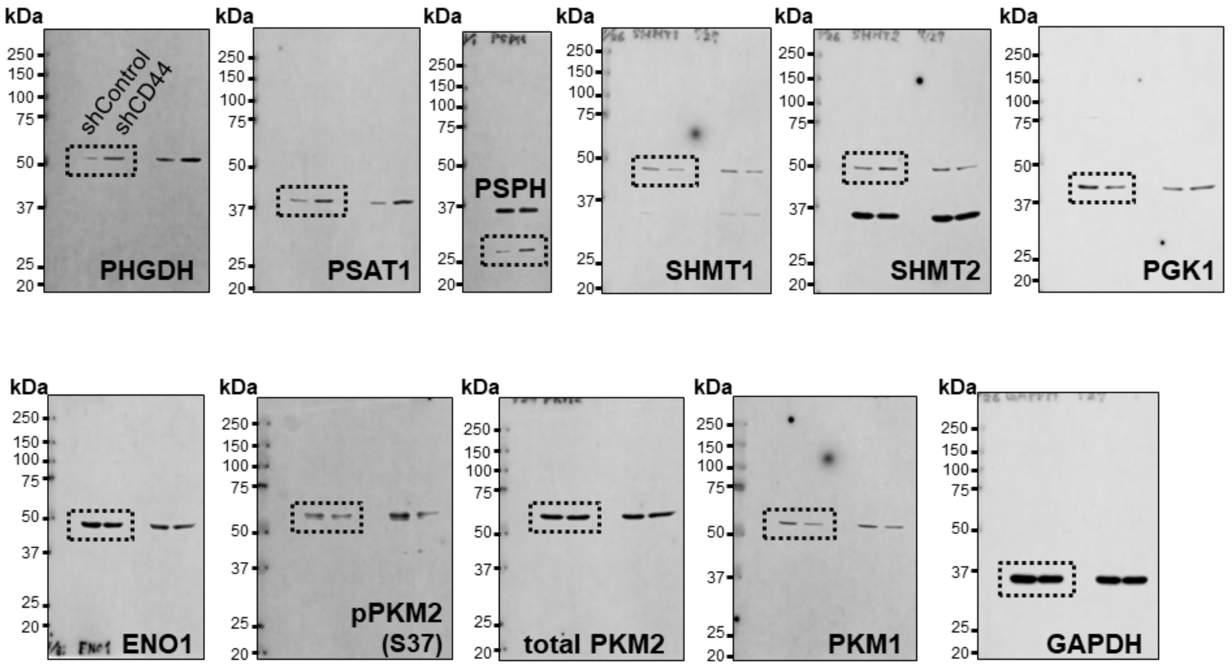

Supplementary Figure 16-2 Uncropped blotting data in **Figure 8d** showing comparison between shControl and shCD44 cells of PHGDH, PSAT1, PSPH, SHMT1, SHMT2, PGK1, ENO1, pPKM2, total PKM2, PKM1 and GAPDH. Dotted squares indicate partial images used in **Figure 8d**.

Suppl. Fig.16 Uncropped blot. data-3

(Suppl. Fig.14a, Part 1)

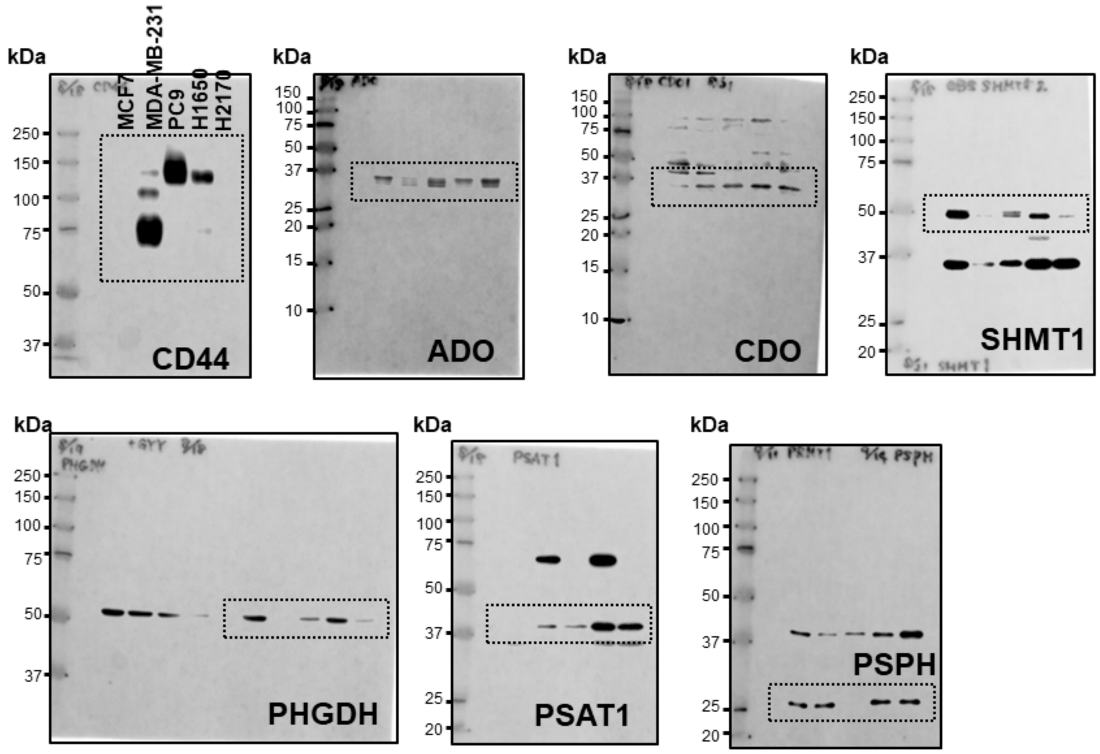

Supplementary Figure 16-3 Uncropped blotting data shown in Part 1 of **Supplementary Figure 14a** showing comparison of CD44, ADO, CDO, SHMT1, PHGDH, PSAT1 and PSPH in different cell lines (MCF7, MDA-MB-231, PC9, H1650, H2170). Dotted squares indicate partial images used in **Supplementary Figure 14a**, Part 1.

Suppl. Fig.16 (continued) Uncropped blot. Data-4

(Suppl. Fig.14a, Part 2)

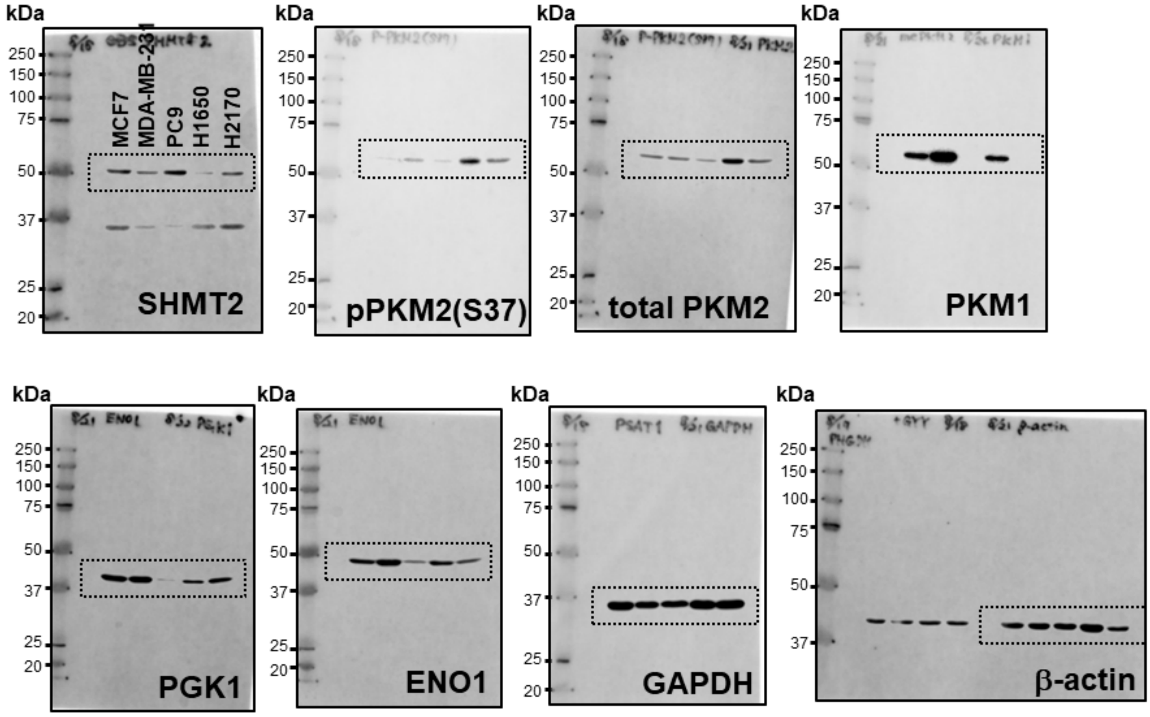

Supplementary Figure 16-4 Uncropped blotting data shown in Part 2 of **Supplementary Figure 14a** showing comparison of SHMT2, pPKM2, total PKM2, PKM1, PGK1, ENO1, GAPDH, and β-actin in different cell lines (MCF7, MDA-MB-231, PC9, H1650, H2170). Dotted squares indicate partial images used in **Supplementary Figure 14a**, Part 2.

Supplementary Table 1 Lists of Raman shifts occurring dominantly in tumours (T-dominant) and those in parenchyma (P-dominant) in the shControl-xenografted NOG mouse liver. These raw data collected from the shControl group (n = 7) were used to establish parent database (DB) depicted in Figure 4 and Suppl. Fig. 3. In these analyses, SERS signals were not accumulated, but processed directly at the smallest detector resolution (~2 cm^{-1}). The shifts with statistical significance ($P < 0.05$) were chosen by unpaired Student's t-test. P mean and T mean indicate mean intensities of the individual Raman shifts at specific wave numbers. The values of dF indicate degree of freedom for unpaired Student's t-test. Resultantly, 6 and 22 different shifts were identified as T-dominant and P-dominant individual shifts, respectively.

cm^{-1}	P mean	T mean	dominant	dF	t-value
289	29.04	42.10	T-dominant	12	-2.354
291	5.76	21.07	T-dominant	12	-4.566
301	72.26	88.22	T-dominant	10	-2.429
464	46.62	56.78	T-dominant	12	-2.239
467	36.84	50.18	T-dominant	12	-2.334
936	57.92	50.96	P-dominant	11	2.367
952	23.62	17.25	P-dominant	9	2.282
956	6.59	1.54	P-dominant	11	2.568
1145	164.83	119.84	P-dominant	10	2.258
1146	170.68	124.13	P-dominant	10	2.649
1148	194.05	145.76	P-dominant	10	2.541
1150	209.99	160.87	P-dominant	10	2.274
1152	204.58	156.50	P-dominant	10	2.531
1154	196.66	150.13	P-dominant	10	2.545
1156	179.13	135.51	P-dominant	10	2.546
1158	152.55	113.26	P-dominant	10	2.355
1160	129.50	93.69	P-dominant	10	2.291
1162	115.58	82.70	P-dominant	10	2.56
1164	107.76	77.65	P-dominant	10	2.683
1166	96.07	69.19	P-dominant	10	2.395
1168	80.47	57.82	P-dominant	9	2.489
1169	76.32	56.87	P-dominant	9	2.912
1171	72.19	55.32	P-dominant	11	2.637
1173	56.30	42.91	P-dominant	10	2.567
1421	1.03	4.96	T-dominant	12	-2.308
1529	42.98	30.29	P-dominant	10	2.411
1534	37.25	23.81	P-dominant	9	2.308
1536	41.11	27.40	P-dominant	9	2.273

Supplementary Table 2 Differences in CE-ESI-MS-based metabolomics of the livers bearing shControl- and shCD44-HCT116-derived tumours.

Metabolites (nmol g^{-1})	**shControl**	**shCD44**	**dF**	**t-value**
**GSH**	**2316 ± 376**	**818 ± 206***	**11**	**3.818**
**GSSG**	**2600 ± 243**	**2124 ± 199**	**11**	**1.504**
**GSH + 2xGSSG**	**7516 ± 372**	**5065 ± 315***	**11**	**4.941**
**Hypotaurine**	**275 ± 40**	**270 ± 35**	**11**	**0.092**
**Taurine**	**11310 ± 626**	**11783 ± 634**	**11**	**0.499**
**ATP**	**1866 ± 133**	**1556 ± 122**	**11**	**1.655**
**ADP**	**697 ± 51**	**886 ± 60**	**11**	**2.179**
**AMP**	**594 ± 61**	**934 ± 41***	**11**	**4.795**
**Energy Charge****	**0.70 ± 0.03**	**0.59 ± 0.01***	**11**	**4.555**
**Adenosine**	**50.7 ± 4.1**	**76.9 ± 9.9**	**11**	**2.002**
**Inosine**	**15.3 ± 3.1**	**18.1 ± 2.7**	**11**	**0.660**
**Hypoxanthine**	**7.1 ± 2.1**	**13.7 ± 1.9***	**11**	**2.253**
**Xanthine**	**11.9 ± 1.4**	**18.7 ± 3.1**	**11**	**1.661**
**Hcy-Hcy**	**3.3 ± 0.4**	**2.5 ± 0.3**	**11**	**1.363**
**Hcy**	**6.4 ± 0.9**	**7.4 ± 1.1**	**11**	**0.627**
**Cys-Cys**	**4.7 ± 0.9**	**2.8 ± 0.4**	**11**	**2.137**
**Cys**	**10.6 ± 1.3**	**9.6 ± 1.3**	**11**	**0.577**
**L-Cysteine sulfinate**	**4.9 ± 2.0**	**3.7 ± 1.4**	**11**	**0.501**
**Met**	**38.9 ± 3.1**	**22.2 ± 0.9***	**11**	**6.308**
**SAM#**	**71.4 ± 1.6**	**63.6 ± 4.4**	**11**	**1.343**
**SAH#**	**14.3 ± 0.7**	**14.7 ± 1.3**	**11**	**0.259**
**Cystathionine**	**41.9 ± 7.4**	**28.0 ± 3.1**	**11**	**1.985**
**Homoserine**	**13.8 ± 5.0**	**8.1 ± 1.8**	**11**	**1.289**
**Spermidine**	**45.9 ± 1.9**	**28.1 ± 1.8***	**11**	**6.452**
**Spermine**	**20.0 ± 1.3**	**13.0 ± 1.6***	**11**	**3.156**
**Glutathione sulfonate**	**11.9 ± 0.9**	**12.5 ± 2.0**	**7**	**0.299**
**Cysteic acid**	**4.1 ± 0.9**	**5.3 ± 1.1**	**7**	**0.850**
**Homocysteic acid**	**4.5 ± 1.0**	**4.4 ± 0.7**	**7**	**0.138**

***P<0.05 versus shControl with unpaired zStudent's t-test. ** Energy charge = (ATP + 0.5 x ADP)/(ATP+ADP+AMP) # SAM and SAH indicate S-adenosyl-L-methionine and S-adenosyl-L-homocysteine, respectively. Values are mean ± SEM. of shControl (n=5) and shCD44 (n=4~8).**

Supplementary Table 3 Lists of Raman shifts occurring dominantly in tumours (T-dominant) and those in parenchyma (P-dominant) in the orthotopic syngeneic mouse glioblastoma model. These raw data collected from the tumour-bearing brain slices (4 separate experiments) were used to build up the database of accumulated lists of the Raman shifts (ALR) depicted in **Figure 4** and **Suppl. Fig. 3**. In these analyses, SERS signals were not accumulated, but processed directly at the smallest detector resolution (~2 cm^{-1}). The shifts with statistical significance ($P < 0.05$) were chosen by unpaired Student's t-test. P mean and T mean indicate mean intensities of the individual Raman shifts at specific wave numbers. The values of dF indicate degree of freedom. Resultantly, 16 and 10 different shifts were identified as T-dominant and P-dominant individual shifts, respectively.

cm^{-1}	P mean	T mean	dominant	dF	t-value
199	53.21	77.92	T-dominant	6	-2.481
202	28.94	51.70	T-dominant	6	-2.697
720	5.81	17.49	T-dominant	4	-3.175
722	12.07	28.33	T-dominant	4	-3.643
724	22.87	42.89	T-dominant	5	-3.138
726	20.95	41.63	T-dominant	5	-2.979
729	6.95	24.88	T-dominant	6	-3.45
731	-4.86	9.51	T-dominant	4	-4.14
733	-15.21	-4.74	T-dominant	5	-2.721
1314	3.20	10.91	T-dominant	3	-3.727
1442	33.44	50.35	T-dominant	6	-2.83
1601	10.81	18.99	T-dominant	6	-2.929
1630	11.66	15.38	T-dominant	6	-2.825
1631	18.55	23.60	T-dominant	5	-3.122
1635	23.72	28.81	T-dominant	5	-2.61
1926	15.54	17.33	T-dominant	6	-2.493
360	-9.32	-14.30	P-dominant	6	3.006
696	36.74	30.24	P-dominant	6	3.711
698	40.07	33.35	P-dominant	5	2.796
702	51.27	42.64	P-dominant	4	3.745
705	42.91	35.65	P-dominant	6	2.521
956	27.10	16.88	P-dominant	6	3.24
958	21.26	10.30	P-dominant	6	2.765
1484	-6.45	-14.37	P-dominant	6	3.582
1488	2.53	-4.24	P-dominant	4	2.863
1804	-9.86	-10.74	P-dominant	6	2.857
